# Population size affected by environmental variability impacts genetics, traits, and plant performance in *Trifolium montanum* L.

**DOI:** 10.1002/ece3.10376

**Published:** 2023-08-07

**Authors:** Kevin Karbstein, Christine Römermann, Frank Hellwig, Kathleen Prinz

**Affiliations:** ^1^ Institute of Ecology and Evolution Friedrich Schiller University Jena Germany; ^2^ Department of Systematics, Biodiversity and Evolution of Plants (with Herbarium) Albrecht‐von‐Haller Institute for Plant Sciences University of Göttingen Göttingen Germany; ^3^ Department of Biogeochemical Integration Max Planck Institute for Biogeochemistry Jena Germany; ^4^ German Centre for Integrative Biodiversity Research (iDiv) Halle‐Jena‐Leipzig Leipzig Germany

**Keywords:** genetic diversity, genetic inbreeding, habitat degradation and fragmentation, intraspecific trait variation, microsatellites, plant performance, population size, *Trifolium montanum*

## Abstract

Population size, genetic diversity, and performance have fundamental importance for ecology, evolution, and nature conservation of plant species. Despite well‐studied relationships among environmental, genetic, and intraspecific trait variation (ITV), the influence of population size on these aspects is less understood. To assess the sources of population size variation, but also its impact on genetic, functional trait, and performance aspects, we conducted detailed population size estimations, assessed 23 abiotic and biotic environmental habitat factors, performed population genetic analyses using nine microsatellite markers, and recorded nine functional traits based on 260 *Trifolium montanum* individuals from 13 semi‐dry grassland locations of Central Europe. Modern statistical analyses based on a multivariate framework (path analysis) with preselected linear regression models revealed that the variation of abiotic factors (in contrast to factors per se) almost completely, significantly explained fluctuations in population size (*R* ^2^ = .93). In general, abiotic habitat variation (heterogeneity) was not affected by habitat area. Population size significantly explained genetic diversity (*N*_A_: *R* ^2^ = .42, *H*_o_: *R* ^2^ = .67, *H*_e_: *R* ^2^ = .43, and *I*: *R* ^2^ = .59), inbreeding (*F*_IS_: *R* ^2^ = .35), and differentiation (*G*_ST_: *R* ^2^ = .20). We also found that iFD_CV_ (ITV) was significantly explained by abiotic habitat heterogeneity, and to a lesser extent by genetic diversity *H*_e_ (*R* ^2^ = .81). Nevertheless, habitat heterogeneity did not statistically affect genetic diversity. This may be due to the use of selectively neutral microsatellite markers, and possibly by insufficient abiotic selective pressures on habitats examined. Small *T. montanum* populations in nonoptimal habitats were characterized by reduced genetic and functional trait diversity, and elevated genetic inbreeding and differentiation. This indicates reduced adaptability to current and future environmental changes. The long‐term survival of small populations with reduced genetic diversity and beginning inbreeding will be highly dependent on habitat protection and adequate land‐use actions.

## Graphical abstract

Despite well‐studied relationships among environmental, genetic, and plant intraspecific trait variation (ITV), the influence of population size on these aspects is less understood. Small mountain clover populations in nonoptimal (niche pessimum) habitats are characterized by reduced genetic diversity and ITV based on functional traits, and increased genetic inbreeding and differentiation. The long‐term survival of small populations will thus be highly dependent on further habitat protection and appropriate land‐use actions.

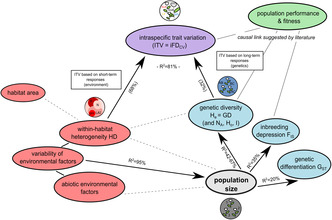

## 1 INTRODUCTION

Relationships among population size, genetic diversity, and performance are of great research interest due to their fundamental importance in plant ecology, evolution, and conservation (De Kort et al., 2021; Leimu et al., 2006; Rosche et al., 2022; Szczecińska et al., 2016). Genetic diversity is generated by DNA mutation and/or meiotic recombination. It provides variation for natural selection to act on, and is, therefore, crucial for evolutionary processes and influences the adaptive potential of species to current and future environmental conditions (Boulding, 2008; Karbstein et al., 2019; Karbstein, Prinz, et al., 2020; Reed & Frankham, 2003). Population size is positively linked to genetic diversity of populations: the larger the population size, the higher the probability of having genetically diverse individuals generated by mutation and recombination, and the lower the consequences of genetic drift. The latter refers to random genetic changes in allele frequencies within populations, which occur, for example, when population size is reduced by habitat destruction or degradation (bottleneck), or dispersal of a few individuals to remote locations (founder effect; reviewed in Freeland et al., 2011).

In small populations, reduced genetic diversity and its negative consequences are frequently observed. They are caused by loss of heterozygosity due to elevated genetic drift (incl. founder and bottleneck effects), and inbreeding depression due to the accumulation of deleterious mutations (Caré et al., 2020; Freeland et al., 2011; Karbstein, Rahmsdorf, et al., 2020; Lynch et al., 1995; Rosche et al., 2022; Schleuning et al., 2009). This leads to reduced performance (i.e., plant function, health, or survival) and fitness (i.e., reproductive output) in small populations, and in the long term, to reduced evolutionary potential to adapt to changing environments and increased risk of extinction (Ellstrand & Elam, 1993; Karbstein, Rahmsdorf, et al., 2020; Leimu et al., 2006; Spielman et al., 2004). In nature, many plant populations are isolated and small, and recent anthropogenic habitat fragmentation further increases isolation and promotes erosion of these populations. However, within plant species, the precise consequences of changes in population size for genetic diversity and inbreeding, as well as performance and fitness of populations, are less understood.

In general, positive relationships between plant population size, genetic diversity, and/or fitness have been inferred, for example, in the Central European rare meadow species *Angelica palustris* (Apiaceae; Dittbrenner et al., 2005), *Arnica montana* (Asteraceae; Duwe et al., 2017; Luijten et al., 2000), *Biscutella laevigata* (Brassicaceae; Rosche et al., 2022), *Dictamnus albus* (Rutaceae; Hensen & Wesche, 2006), *Pulsatilla* (Ranunculaceae; Hensen et al., 2005; Szczecińska et al., 2016), or *Trifolium montanum* (Fabaceae; Karbstein, Prinz, et al., 2020). These examples are supported by overall mean relationships across species (reviewed in Leimu et al., 2006). Such positive relationships are likely causal and exist for two main reasons: As noted above, first, a reduction in population size decreases genetic diversity, increases inbreeding depression, and lowers plant fitness and population growth rate, resulting in a further decrease in population size (“vortex of extinction”; Ellstrand & Elam, 1993; Leimu et al., 2006; Rosche et al., 2022). Second, variation in habitat quality, age, and structure across the species' distribution range can also affect these relationships. For instance, suitable habitat quality (niche optimum) typically leads to large populations with high growth rates, whereas poor habitat quality (niche pessimum) leads to small populations with low growth rates, resulting in changes in genetic diversity, performance, and fitness (Leimu et al., 2006; Karbstein, Prinz, et al., 2020; Reisch et al., 2021).

These thoughts are also apprehended and summarized by the “abundant center hypothesis” (ACH; Sagarin et al., 2006; Sagarin & Gaines, 2002). The ACH predicts the largest population size along with the highest genetic diversity, performance, and fitness for niche‐optimum (range center) populations, but a decline towards niche‐pessimum (range edge) populations due to decreasing habitat quality, and effects of genetic drift, restricted gene flow, and inbreeding as well as increasing genetic differentiation (Brown, 1984; Hampe & Petit, 2005; Hardie & Hutchings, 2010; Hirsch et al., 2015; Hoffmann & Blows, 1994; Wagner et al., 2012). However, highly genetically differentiated, niche‐pessimum/marginal range populations may still have sufficient genetic variation and can be valuable sources and important targets for nature conservation efforts due to site‐specific adaptations (Karbstein, Prinz, et al., 2020; Kirschner et al., 2020). General relationships also depend on several other factors, such as plant breeding system, life history, and species rarity, but also on the use of neutral or selective genetic markers (reviewed in Angeloni et al., 2011; Hamrick et al., 1979; Reed & Frankham, 2003; Reisch & Bernhardt‐Römermann, 2014; Spielman et al., 2004). Nevertheless, detailed intraspecific population size measurements affected by local, comprehensive abiotic and biotic records based on sufficient replications are often missing for single plant species, attributable to high sampling efforts and a lack of suitable model systems. Consequently, the intraspecific link between population size, genetics and fitness to the local environment is not sufficiently understood.

In addition to well‐observed “traditional” fitness measures such as height, number of shoots/leaves, flowers, or seeds, or germination rates (e.g., Karbstein, Rahmsdorf, et al., 2020; Rosche et al., 2022; Syngelaki et al., 2020), modern ecological research also focuses on the link between morpho‐physio‐phenological traits and plant performance and fitness, called “functional traits” (Nock et al., 2016; Violle et al., 2007). Some functional traits address plant form (e.g., releasing height “RH,” or leaf area “LA”), while others capture plant physiology and function (e.g., specific leaf area “SLA,” or performance index “PI”; see also Díaz et al., 2016). Functional traits are often used to explain individual but also population and ecosystem responses related to environmental conditions and changes such as habitat fragmentation or climate change (Bernhardt‐Römermann et al., 2011; Bucher et al., 2016; Karbstein et al., 2019; Römermann et al., 2009; Westerband et al., 2021). They are strongly dependent on local abiotic soil and climatic factors and biotic competition, are highly species‐specific, and thus should be studied for each model system.

Variation of functional traits is initially measured as phenotypic plasticity, that is, the ability of a single genotype to express different phenotypes depending on its abiotic and biotic environment (Gratani, 2014; Sultan, 2000). Phenotypic plasticity has an (epi)genetic basis and contributes to genetic differentiation and speciation processes (Agrawal, 2001; Westerband et al., 2021). Genotype‐dependent plasticity of individuals results in phenotypic variation of a given plant population. Observations in natural plant populations have shown that phenotypic and genetic variation is associated with each other, particularly with respect to morphology‐related (Karbstein, Prinz, et al., 2020; Waitt & Levin, 1998) but also ecological or ecophysiologically important traits (Ackerly et al., 2000; Hughes et al., 2019; Locascio et al., 2009; Via et al., 1995). In the semi‐dry grassland species *T. montanum* (mountain clover), intraspecific trait variation (ITV) based on functional traits was significantly positively associated with environmental habitat heterogeneity and genetic diversity of populations (Karbstein, Prinz, et al., 2020). Though abiotic habitat heterogeneity was predominantly responsible for ITV, both aspects are important to consider when studying the consequences for plant performance under present and changing environmental conditions (Karbstein et al., 2019; Karbstein, Prinz, et al., 2020). Despite the aforementioned study inferred positive mean relationships, the influence of population size on these relationships remains unobserved to date.

*Trifolium montanum* populations in (semi‐)dry calcareous grasslands of Central Europe are well suited to fill the gaps of knowledge. Formerly, *T. montanum* was widespread, but its abundance in Central Europe declined during the last decades due to habitat degradation and fragmentation, and today the species is regionally threatened (Breunig & Demuth, 1999; Garve, 2004; Matter et al., 2012). Strategies for the protection and management of (semi‐)dry grasslands and their endangered species continue to be a hot topic for both theorists and practitioners involved in conservation biology. Therefore, in this study, we aim to analyze relationships among population size, environment, genetic diversity and inbreeding, and population performance based on functional traits regarding the herbaceous, calcareous (semi‐)dry grassland species *T. montanum*. We addressed the following questions: Do small and large *T. montanum* populations differ in their (1) abiotic and biotic environments, in their (2) functional trait characteristics, and (3) genetic diversity, inbreeding, and differentiation? And (4) how population size affected by environmental factors impacts genetic features and intraspecific trait variation (ITV)? Results will subsequently be discussed in the context of long‐term viability and nature conservation of *T. montanum* populations.

## 2 MATERIALS AND METHODS

### 2.1 Study species

Mountain clover (*Trifolium montanum* L., Fabaceae) is distributed across Eurasia; it is a diploid (2*n* = 16), perennial, up to 70 cm tall herb of extensively used grasslands (GBIF Secretariat, 2017; Jäger, 2011; Klotz et al., 2002; Rice et al., 2015; Schleuning & Matthies, 2008). In Germany, this species inhabits (semi‐)dry calcareous *Festuco‐Brometea* grasslands but also occurs along shrub and forest edges and way‐ and roadsides (Karbstein, Prinz, et al., 2020). Denticulate leaflets with silky undersurface and flocculent‐haired shoots bearing up to six flower heads characterize this species (Jäger, 2011; Schleuning et al., 2009; Schleuning & Matthies, 2008). A flower head comprises about 150 zygomorphic flowers with one ovule (Matter et al., 2013; Schleuning et al., 2009). Sweet‐smelling, yellowish‐white flowers attract pollinators such as bees, butterflies, and small beetles (Jäger, 2011; Schleuning et al., 2009; Schleuning & Matthies, 2008; pers. obs.). Mountain clover is predominantly outcrossing, and pollination occurs from May to July; the mean number of seeds per fruit head varies considerably between populations, probably due to pollinator failure in small populations (Schleuning & Matthies, 2008).

Seed dispersal starts in July and mainly occurs on a regional scale (Schleuning et al., 2009; Schleuning & Matthies, 2008). In the course of this study, we observed local grazing by sheep, goats, cattle, and horses, suggesting endozoochorous, geographically restricted seed dispersal in Central Germany. Vegetative reproduction has been frequently observed in *T. montanum* (Klimeš & Klimešová, 1999; Klimešová et al., 2017; Klimešová & Bello, 2009). In this study, 30% of the sampled *T. montanum* individuals showed clonality in the form of epigeogenous stems and root splitters. Clones are connected and/or are growing very close to mother plants, and were easily sorted out beforehand. We also found no evidence of sampled clones within populations across the dataset (see assessment of population genetics below). In addition, the main root varies remarkably in thickness and length (Figure S1a,b). Among study locations, we observed a maximum diameter and length of approximately 20 mm and 20 cm, respectively, presumably due to age‐related and/or environmental effects. For example, an up to 30‐year‐old individual was observed, and far older ones are expected (Figure S1b).

### 2.2 Study locations, estimation of population size, and sampling

**FIGURE 1:**
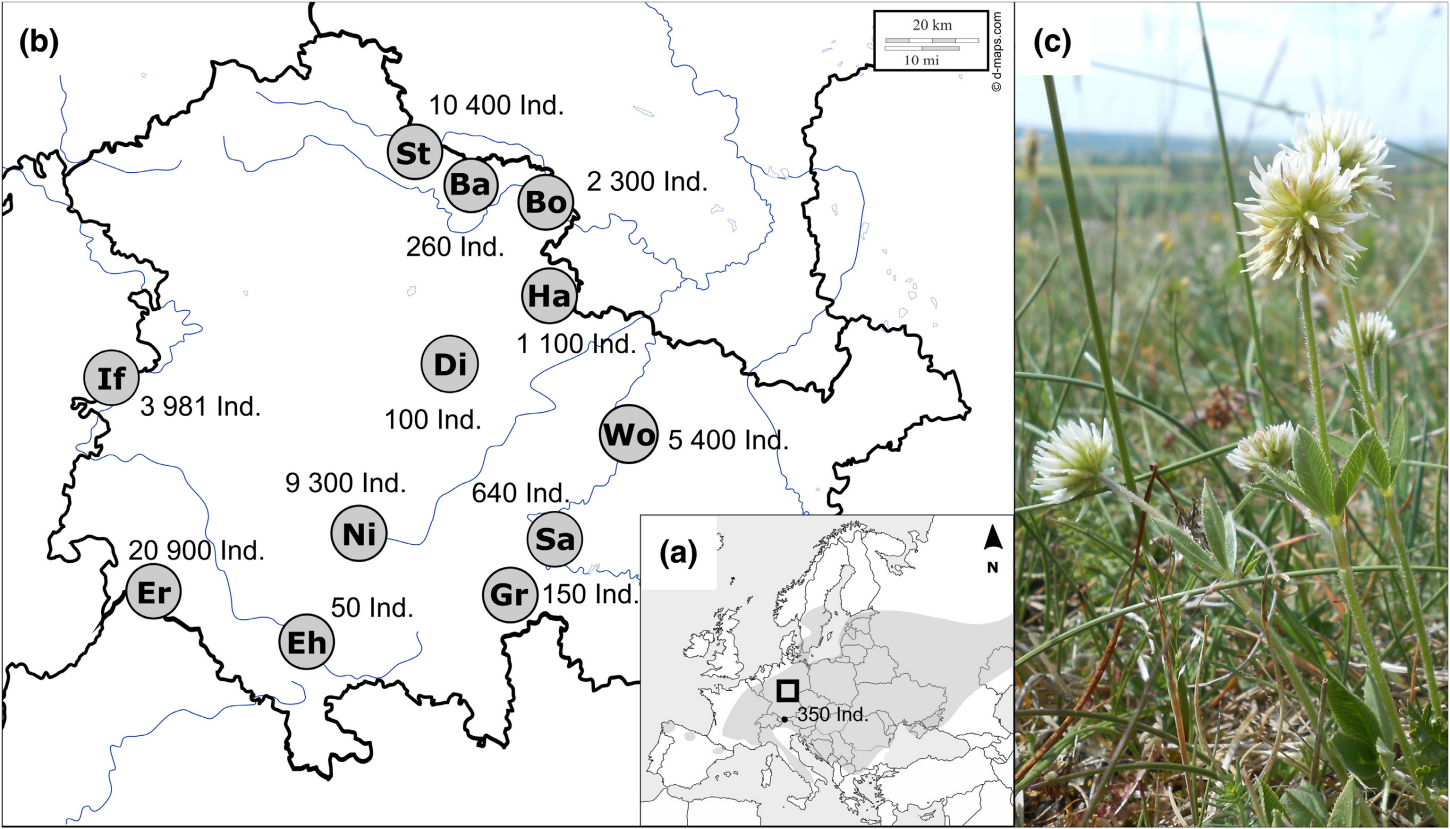
(a) European distribution range of *Trifolium montanum* (mountain clover) highlighted in gray according to Meusel and Jäger (1998). The black square indicates the sampling area in Germany. The black dot shows the sampling location in Austria (“KW”). (b) Sampling localities in Central Germany (see Table 1 for abbreviations and further information). Black circles highlight sampling locations. Numbers near sampling locations indicate the population sizes (i.e., number of individuals per population). Black lines illustrate the borders of the German federal states (focus on Thuringia). The figures (a,b) were taken from Karbstein, Prinz, et al. (2020) (published under Creative Commons License) and modified here. The original maps were downloaded from d‐maps.com. (c) *Trifolium montanum* population on dry grasslands near Bottendorf ("Bo"). Mountain clover has whitish, zygomorphic flowers, and shoots with three denticulate leaflets. Image source: Kevin Karbstein.

We sampled 13 locations in Central Europe, covering a wide range of environmental conditions (Table 1, Figure 1, see also Karbstein, Prinz, et al., 2020). To estimate population size at each location, we carried out two different strategies. The number of individuals was counted if populations contained less than 500 individuals and rounded to the nearest 10, or extrapolated by averaging the number of individuals from 15 to 20, 4 m^2^‐records (individuals per m^2^, abundance) multiplied by the area occupied by a population (recorded with GPX‐tracks, Figure S2) and rounded to the nearest 100, following the approach of Hensen et al. (2005).

**TABLE 1:** Sampling localities of *Trifolium montanum* populations in Central Germany and Austria (KW; see also Karbstein, Prinz, et al., 2020).

Location	Date	Lat. (N)	Long. (E)	Habitat area (m^2^)	Abundance (Ind. m^−2^)	Population size
Riezlern (KW)	17.07.2015	47.361036	10.173825	285	1.23	350 (small)
Bottendorf (Bo)	22.05.2016	51.316042	11.396525	2101	1.12	2300 (large)
Hardisleben (Ha)	25.05.2016	51.162917	11.446789	1249	0.92	1100 (small)
Jena‐Wogau (Wo)	29.05.2016	50.924306	11.665083	959	5.63	5400 (large)
Bad Frankenhausen (Ba)	31.05.2016	51.367267	11.103056	197	1.34	260 (small)
Steinthaleben (St)	05.06.2016	51.409550	11.004850	1394	7.45	10,400 (large)
Saalfeld (Sa)	08.06.2016	50.631003	11.383729	452	1.41	640 (small)
Ifta (If)	12.06.2016	51.086633	10.148017	3981	2.03	8100 (large)
Niederwillingen (Ni)	15.06.2016	50.776294	11.027711	951	9.73	9300 (large)
Dielsdorf (Di)	19.06.2016	51.0952330	11.188406	23	4.06	100 (small)
Erbenhausen (Er)	23.06.2016	50.565556	10.157383	4627	4.52	20,900 (large)
Großneundorf (Gr)	28.06.2016	50.532456	11.294961	174	0.87	150 (small)
Ehrenberg (Eh)	29.06.2016	50.478583	10.665786	20	2.26	50 (small)

*Note*: Details include sampling locality names, sampling dates, decimal latitude (north) and longitude (east) in WGS84, and calculated habitat area, frequency, and population size (classified as small or large, see details in Section 2.3).

A “population” was defined as a group of individuals of the same species separated by their closest conspecifics by at least 100 m or by natural barriers such as agricultural areas or forests (Bachmann & Hensen, 2006). We collected 20 *T. montanum* individuals for functional trait and population genetic analyses at each location (sampling points were distributed equally within a habitat). In total, we sampled up to 20 individuals per population and 260 individuals in Riezlern (KW) in July 2015 and in Central Germany from May to June 2016.

### 2.3 Assessment of habitat characteristics, population genetics, and functional traits

As described in Karbstein, Prinz, et al. (2020), data preprocessing and filtering of environmental factors and functional traits were done with R in order to remove outliers or collinearities (see also Dormann et al., 2013), and manual editing of microsatellite marker raw data was performed to remove ambiguous scoring results. In this study, we additionally evaluate comprehensive biotic vegetation record data, habitat area estimates, and population sizes (additionally classified as “small” and “large” at the median of the distribution; small = 50 to 1100 individuals, and large = 2300 to 20,900 individuals) together with the previously analyzed environmental, genetic, and functional trait variables.

To characterize the environmental conditions per location (Table 1), we conducted a maximum of five vegetation records (2 m × 2 m = 4 m^2^; except three in KW and four in Eh), resulting in a final sample size of 64 records. The Schmidt scale (+, 1, 3, 5, 8, 10, 15, 20, 25, 30, 40, 50, 60, 70, 80, 90, and 100; Schmidt, 1974) was used to estimate plant species abundance. Within vegetation records, we noted cover percentages of herb layer and bare ground as well as crop height (95% of stand height). Additionally, within each vegetation record, we recorded GPS coordinates, altitude, slope exposure, slope, leaf area index (LAI), soil depth, and soil characteristics. LAI is predominantly treated here as an abiotic factor, as it indicates the light availability of *T. montanum* plants surrounded and shaded by taller grass species. Soil samples were characterized by cation‐exchange capacities in total (CEC_pot_), for sodium (CEC_Na_), potassium (CEC_K_), calcium (CEC_Ca_), and magnesium (CEC_Mg_), and soil reaction with de‐ionized water (pH_H2O_ or pH) and potassium chloride (pH_KCl_), organic carbon (C_org_), lime (CaCO_3_), nitrogen (N), plant available phosphor (P), and plant available potassium (K; mg/100 g; CEC after DIN ISO 13536 with flame atomic absorption spectrometry; N, P, and K with calcium acetate lactate method). Mean annual temperature (*T*_a_) and mean annual precipitation (*P*_a_) were calculated by interpolation using ArcMap vers. 10.5 (ESRI Inc.) and data derived from WorldClim 1.4 global climate database from 1960 to 1990 (www.worldclim.org; Hijmans et al., 2005).

We estimated the abundance of *T. montanum* individuals as the mean of cover percentages derived from vegetation records. Species richness (S) and evenness (E; Heip et al., 1998) were calculated using the “vegan” R package vers. 2.6‐4 (Oksanen et al., 2022). Evenness is 0 if a species completely dominates vegetation records, and 1 if all species are equally abundant. For each vegetation record, we generated community‐weighted Ellenberg indicator values (Ellenberg et al., 2001) of light availability (wL), climatic continentality (wK), soil humidity (wF), temperature (wT), soil acidity (wR), and soil fertility (wN) as the sum of species abundance multiplied with its indicator value (Bartelheimer & Poschlod, 2016; Diekmann, 2003; Ellenberg et al., 2001). All environmental factors are listed in Table 2. We also used the calculated abiotic within‐habitat heterogeneity (HD) as mean coefficient of variation (CV, ratio of the standard deviation to the mean) based on nonautocorrelated environmental factors from Karbstein, Prinz, et al. (2020): altitude (CV_altitude_), slope exposure (CV_slope exposure_), slope (CV_slope_), leaf area index (CV_LAI_), soil depth (CV_soil depth_), soil cation‐exchange capacity (CV_CECpot_), pH (CV_pH_), soil nitrogen content (CV_N_), soil phosphor content (CV_P_), and soil potassium content (CV_K_; Table 2; see also Tables 1 and 2 in Karbstein, Prinz, et al., 2020).

**TABLE 2:** Mean abiotic and biotic environmental factors of 13 *Trifolium montanum* locations in Central Europe (see also Karbstein, Prinz, et al., 2020).

Location	Altitude* (m.a.s.l.)	*T*_a_ (°C)	*P*_a_ (mm)	Slope* (°)	Slope exp.*	LAI*	Crop height (cm)	Soil depth* (cm)	CEC_pot_* (cmol /kg)	pH*	N* (%)	P* (mg/100 g)	K* (mg/100 g)	wL	wK	wF	wT	wR	wN	S	E	Cover Herb (%)	Bare Ground (%)	HD
KW	1057	5.7	1045	2.0	NW	5.0	‐	16.8	12.9	6.1	0.57	0.7	3.6	6.7	3.6	5.5	4.2	6.8	4.1	31	0.82	97	1	0.143
Bo	180	8.7	511	3.0	NE	1.2	11	10.9	15.6	5.6	0.36	0.8	5.2	7.9	3.3	3.3	5.5	7.7	2.8	14	0.79	91	8	0.303
Ha	233	8.4	548	12.7	W	2.1	23	14.6	16.0	6.7	0.41	1.3	20.0	7.4	4.5	3.8	5.6	7.5	3.3	20	0.73	94	5	0.235
Wo	298	7.8	628	6.6	W	2.4	59	13.6	15.9	7.7	0.42	1.2	23.8	7.6	3.3	3.4	5.4	7.9	3.1	28	0.73	89	5	0.257
Ba	260	8.2	565	18.3	W	1.9	56	21.5	14.8	5.4	0.61	0.9	8.6	7.6	3.9	3.1	5.5	7.9	2.6	19	0.68	81	10	0.244
St	265	8.2	567	9.1	NE	1.2	46	19.0	16.3	7.6	0.58	1.4	7.0	7.7	3.6	3.1	5.5	7.9	2.7	24	0.66	95	3	0.357
Sa	332	8.1	598	5.5	NW	1.9	39	16.6	15.7	8.0	0.59	2.0	12.4	7.5	3.8	3.5	5.3	7.6	3.0	20	0.60	86	8	0.325
If	358	7.7	709	7.2	SW	2.4	51	17.7	16.8	7.9	0.37	0.6	16.0	7.7	3.0	3.3	5.3	7.8	2.9	29	0.65	94	5	0.150
Ni	366	7.7	588	16.5	N	4.1	36	15.8	17.5	7.8	0.61	1.7	25.2	7.8	3.4	3.2	5.3	7.8	2.8	24	0.68	98	2	0.169
Di	232	8.3	535	7.9	NW	4.4	83	21.0	16.6	7.5	0.41	1.0	14.6	7.4	4.0	3.8	5.5	7.5	4.6	33	0.83	99	1	0.295
Er	546	6.9	767	10.2	W	5.8	57	9.6	16.3	7.7	0.72	2.6	24.4	7.7	3.6	3.3	5.4	7.8	2.7	29	0.80	97	2	0.346
Gr	540	6.7	734	16.1	S	2.4	23	11.0	14.8	7.3	0.83	0.9	6.0	7.6	3.4	3.5	5.3	7.5	3.1	30	0.74	96	3	0.151
Eh	499	7.4	661	4.9	N	3.5	45	10.9	16.7	7.8	0.75	2.4	28.0	6.9	4.2	4.0	5.3	7.5	4.1	19	0.69	76	0	0.193
Statistics	*F* = 1.1, ***	*F* = 6.2, ***	*F* = 6.2, ***	*F* = 4.7, ***	*χ* ^2^ = 43, ***	*F* = 21, ***	*F* = 7.2, ***	*F* = 15 ***	*F* = 7.5, ***	*F* = 21, ***	*F* = 11, **	*χ* ^2^ = 41, ***	*F* = 24, ***	*F* = 5.2, ***	*F* = 4.0, ***	*F* = 22, ***	*F* = 12, ***	*F* = 3.9, ***	*F* = 9.7, ***	*F* = 9.6, ***	*F* = 2.3, *	*χ* ^2^ = 38, ***	*χ* ^2^ = 32, **	

*Note*: Medians were calculated for exposition (indicated by letters N = North, E = East, S = South, W = West) and community‐weighted Ellenberg indicator values of light availability (wL), climatic continentality (wK), soil humidity (wF), temperature (wT), soil acidity (wR), and soil fertility (wN), and cover percentages of herb layers and bare ground. *=Abiotic environmental factor used to calculate habitat heterogeneity (HD) in Karbstein, Prinz, et al. (2020). See cited Dryad data repository in Section (Data Availability Statement) for environmental raw data. Statistics were performed to infer location‐dependent differences among environmental factors (***p < .001, **p < .01, see Section 2.4).

Abbreviations: CEC_pot_, potential cation‐exchange capacity; E, evenness; exp., slope exposure; K, soil potassium content; N, soil nitrogen content; P, soil phosphor content; *P*_a_, annual precipitation; S, species richness; *T*_a_, annual temperature.

Subsequently, we performed population genetic laboratory work procedures and recorded microsatellite information for each population. Traditional microsatellite markers are highly efficient to assess population genetic diversity and inbreeding (reviewed in Hodel et al., 2016; Rosche et al., 2022). DNA extraction was done using CTAB protocol (Doyle & Doyle, 1987; Saghai‐Maroof et al., 1984; modified here), nine microsatellite loci were applied based on the protocol of Matter et al. (2012), locus‐specific touchdown PCRs were conducted (Korbie & Mattick, 2008), followed by fragment length analyses and scoring for population genetic analyses (Figures S3‐S5). In total, 255 individuals characterized by at least four microsatellite loci were genotyped. We inferred a mean loci coverage of 90% per individual, that is, on average, 90% of loci were present in a single individual. Missing data are evenly distributed across populations of the dataset (see Dryad data repository in Section [Data Availability Statement]), and thus no bias in genetic analyses is expected. Individuals across populations belong to the same genetic lineage, as inferred by principal coordinate analyses (PCoAs) based on Nei's genetic distances in GenAlEx and Structure vers. 2.3.4 (Pritchard et al., 2000) analyses in Karbstein, Prinz, et al. (2020). In addition, we performed a PCoAs based on Nei's genetic distances to ensure that sampled individuals within populations did not represent clones that would bias genetic diversity and differentiation indices (Figure S6).

We calculated allelic richness (*N*_A_, number of alleles), private allelic richness (*P*_Ap_, number of private alleles), observed (*H*_o_) and expected heterozygosity (*H*_e_), and Shannon's diversity index (*I*), inbreeding coefficient (*F*_IS_), and differentiation of a single population relative to all populations (*G*_ST_) using GenAlEx vers. 6.503 (Table 3; Hardy, 1908; Nei, 1973; Nei, 1978; Peakall & Smouse, 2006; Peakall & Smouse, 2012; Shannon, 1948; Weinberg, 1908; Wright, 1950). *H*_o_ reflects the ratio of heterozygotes to homozygotes, and *H*_e_ indicates the ratio of heterozygotes expected under Hardy–Weinberg equilibrium (HWE) to homozygotes in a given population (*H*_o_ = *H*_e_, if the population is in HWE); *I* also indicates genetic diversity, but this parameter is not limited to −1/1, making it highly appropriate in cases of large diversity differences. *N*_A_ is the simplest genetic index, but unlike other indices, it depends strongly on population history (e.g., larger populations with past bottleneck events may have high *H*_e_, *H*_o_, and *I* but low *N*_A_); *F*_IS_ depends on the ratio of *H*_o_ and *H*_e_ and indicates homozygote excess (i.e., inbreeding, >0), or heterozygote excess (<0), and *G*_ST_ indicates the genetic distinctiveness of a given population relative to all examined populations (see also Freeland et al., 2011). In Karbstein, Prinz, et al. (2020), *H*_e_ was applied as genetic diversity measure (GD) and proved to be the best index to explain the relationships among trait variation, habitat heterogeneity, and genetic diversity *in T. montanum*. Therefore, *H*_e_ as GD was also used here.

**TABLE 3:** Mean genetic properties of 13 *Trifolium montanum* populations in Central Europe using nine microsatellite markers (see also Karbstein, Prinz, et al., 2020). Abbreviations of locations are explained in Table 1.

Location	*N*	*n*	*N*_A_	P_Ap_ (%)	*H*_o_	*H*_e_ (GD)	*I*	*F*_IS_	*G*_ST_
KW	350	20	52	5.770	0.533	0.597	1.251	0.107	0.067
Bo	2300	20	63	1.590	0.604	0.612	1.343	0.013	0.054
Ha	1100	20	71	5.630	0.594	0.630	1.450	0.057	0.042
Wo	5400	20	68	4.410	0.654	0.654	1.460	0.000	0.035
Ba	260	19	53	0.000	0.560	0.666	1.384	0.159	0.049
St	10,400	19	63	1.590	0.662	0.686	1.472	0.035	0.060
Sa	640	20	56	3.570	0.622	0.637	1.347	0.024	0.056
If	8100	18	59	1.690	0.667	0.678	1.465	0.016	0.032
Ni	9300	20	64	4.690	0.630	0.661	1.473	0.047	0.041
Di	100	20	56	0.000	0.607	0.647	1.369	0.062	0.042
Er	20,900	20	59	5.080	0.690	0.658	1.419	−0.049	0.042
Gr	150	19	49	0.000	0.531	0.570	1.185	0.068	0.059
Eh	50	20	52	3.850	0.575	0.595	1.241	0.034	0.056

*Note*: We did not calculate location‐wise differences (e.g., as done in Tables 2 and 4) because genetic indices are based on different independent loci instead of true replications. For microsatellite raw data, see Dryad data repository cited in Section (Data Availability Statement).

Abbreviations: *F*_IS_, inbreeding coefficient; *G*_ST_, differentiation of a given subpopulation relative to all populations; *H*_e_, expected heterozygosity (used as diversity index GD in Karbstein, Prinz, et al., 2020; *H*_o_, observed heterozygosity; *I*, Shannon's diversity index; *N*, population size, *n*, sample size; *N*_A_, allelic richness; *P*_Ap_, private allelic richness.

We then assessed the following functional traits based on 260 individuals in 13 populations: Releasing height (RH), total dry aboveground biomass (AGB), leaf area (LA), specific leaf area (SLA), leaf dry matter content (LDMC), the ratio of variable fluorescence to maximum fluorescence (F_v_/F_m_), performance index on absorption basis (PI), stomatal pore surface (SPS), and stomatal pore area index (SPI; Balasooriya et al., 2009; Cornelissen et al., 2003; Pérez‐Harguindeguy et al., 2013; Sack et al., 2003; Strasser et al., 2000; Strasser et al., 2004). By sampling only flowering to early fruiting individuals, we ensured the comparability of functional traits among the populations studied (see also Römermann et al., 2016). All functional traits are listed in Table 4. Finally, we used the mean CV of all traits as intraspecific functional trait variation (iFD_CV_). This measure focuses on trait variation rather than trait differences, captures trait space and variation, and is suitable for studying environmental and genetic effects on trait variation (Karbstein, Prinz, et al., 2020).

**TABLE 4:** Mean functional traits and intraspecific functional trait variation (iFD_CV_) of 13 *Trifolium montanum* populations in Central Europe (see also Karbstein, Prinz, et al., 2020).

Location	RH (cm)	AGB (g)	LA (mm^2^)	SLA (mm^2^ mg^−1^)	LDMC (mg g^−1^)	F_v_/F_m_	PI	SPS (μm^2^)	SPI	iFD_CV_
KW	52.1	0.91	891	16.4	270.5	*–*	–	260.3	2.95	0.173
Bo	12.3	0.28	237	17.1	269.9	0.841	9.45	230.5	3.35	0.228
Ha	23.7	0.96	641	15.8	272.4	0.847	11.75	255.3	2.64	0.205
Wo	34.4	2.02	1071	17.0	255.7	0.841	7.91	239.9	2.96	0.210
Ba	25.8	0.68	359	16.9	274.5	0.838	10.16	254.4	2.92	0.196
St	23.8	0.39	296	17.3	292.2	0.830	7.80	259.6	2.88	0.265
Sa	31.4	0.96	922	15.6	277.8	0.839	9.91	237.3	2.73	0.242
If	39.0	1.14	684	15.0	289.9	0.839	7.21	251.5	2.59	0.184
Ni	53.6	1.57	1332	14.0	268.6	0.841	9.21	275.8	2.83	0.204
Di	47.8	1.22	430	16.1	268.8	0.825	6.32	282.0	3.52	0.202
Er	42.2	1.28	743	16.7	264.9	0.846	8.50	243.7	2.59	0.224
Gr	27.5	0.86	855	17.7	262.5	0.848	10.20	266.3	2.53	0.149
Eh	52.9	1.10	1260	16.5	251.1	0.841	10.40	266.8	2.53	0.153
Statistics	*F* = 57, ***	*χ* ^2^ = 121, ***	*χ* ^2^ = 180, ***	*F* = 6.1, ***	*F* = 7.5, ***	*F* = 5.9, ***	*F* = 5.0, ***	*F* = 5, ***	*F* = 8.6, ***	

*Note*: All functional traits listed here were used to calculate iFD_CV_. Abbreviations of locations are explained in Table 1

See the cited Dryad data repository in Section (Data Availability Statement) for trait raw data. Statistics were performed to infer location‐wise differences among functional traits (***p < .001; see Section 2.4).

Abbreviations: AGB, total dry aboveground biomass; F_v_/F_m_, variable/maximum fluorescence; LA, leaf area; LDMC, leaf dry matter content; PI, performance index; RH, releasing height; SLA, specific leaf area; SPI, stomatal pore area index; SPS, stomatal pore surface.

### 2.4 Statistical data analyses

Statistical analyses were performed using R vers. 4.0.1 (R Core Team, 2022). We calculated mean values for numerical variables, medians for ordinal variables (slope exposure and indicator values), and CVs for diversity variables (iFD_CV_, HD) as the ratio of standard deviation to mean. We applied QQ plots to test for normal distribution, but additionally performed Shapiro–Wilk tests when results were inconclusive. To infer location‐/population‐wise differences in environmental factors, genetic indices, or functional traits, we conducted analyses of variance (ANOVA; “*F*”), or Kruskal–Wallis tests (*H* test, ʻ*χ* ^2^ʼ) when data normality and homoscedasticity were not fulfilled. We then used either Tukey‘s HSD test or pairwise Wilcoxon rank‐sum tests (Holm correction) to examine differences between specific groups within a variable. In separate multiple linear regression models, variable contribution of scaled variables was extracted from the models by using the ratio of the respective variable estimate to the sum of the total estimates.

To infer whether small and large *T. montanum* populations differ in their environmental and functional traits (Research Questions 1, 2), we performed principal component analyses (PCAs; Hotelling, 1933; Pearson, 1901) using standardized (zero mean, unit variance) and range‐shifted (adding +5 to all values) environmental factors and functional traits. Riezlern (KW) was excluded from multivariate analyses because of its significantly different biotic and abiotic conditions compared to Central German locations (alpine vs. subcontinental climate), which made it impossible to study general environmental differentiation (PCAs not shown). To investigate environmental differentiation among populations, we ran a PCA based on environmental factors only (axis lengths < 1). Second, we ran a PCA based on functional traits (axis lengths < 1), and correlated the PCA axes with environmental factors (1000 permutations) and only showed the significant factors (*p* < .05). Populations were highlighted according to size to examine their environmental and trait (dis)similarity. In addition, we correlated each PCA axis with population size, and significant PCA axes with environmental factors or traits, to elucidate the most important features separating small and large populations.

To infer associations among population size and genetics, and among population size, environment, genetics, and traits (Research Questions 3, 4), and to only include relevant relationships in the subsequent multivariate framework (path analysis), we conducted linear regression models (LMs). We used logarithmized population size and habitat area to achieve linearity, and specified the following main formulas: (i) Population size explained by single nonautocorrelated habitat factors (altitude, slope exposure, slope, LAI, soil depth, CEC_pot_, pH, N, P, K); (ii) HD explained by habitat area; (iii) population size explained by single nonautocorrelated habitat heterogeneity factors (CV_altitude_, CV_slope exposure_, CV_slope_, CV_LAI_, CV_soil depth_, CV_CECpot_, CV_pH_, CV_N_, CV_P_, CV_K_) and HD; (iv) population genetic indices (*N*_A_, *P*_Ap_, *H*_e=_GD, *H*_o_, *I*, *F*_IS_, *G*_ST_) explained by population size. We ensured that the diversity variables used are saturated, that is, that enough individuals per population were sampled to obtain a good diversity estimate of the entire population (see Figures S6–S8 in Karbstein, Prinz, et al., 2020). In addition, spatial autocorrelation among populations/habitats with respect to HD, GD, and iFD_CV_ was checked before using Moran's *I* values (Moran, 1950), and found to be very weak (Karbstein, Prinz, et al., 2020). Therefore, we did not run specialized LMs accounting for spatial autocorrelation. For multiple linear regression models, we standardized explanatory variables to zero mean and unit variance. Simplification was conducted using the backward selection method: we always excluded the least significant variable (*p* > .1) until we reached the final model (Crawley, 2015). Then, we carried out ANOVAs and additionally calculated the Akaike information criterion (AIC) to control each simplification step. We checked the final models for normality, homoscedasticity, and linearity.

Relationships among population size, environment, genetics, and trait variation are complex. Variables can be used as response, and in other relationships as explanatory variables. For example, habitat factors explain population size, but population size explains genetic indices, and genetic diversity indices explain trait variation, etc. We therefore set up a multivariate environment using a structural equation model (local SEM, path analysis). We only included previously significant relationships (see Section 3) and did not include interactions between explanatory variables to avoid overcomplicating the modeling and interpretation of results. We specified the following formulas: (ii) Population size explained by single nonautocorrelated variation of habitat factors (except for nonsignificant CV_CECpot_, CV_slope_) and HD; (iii) HD explained by habitat area; (iv) population genetic diversity indices (except for nonsignificant *P*_*Ap*_) explained by population size, and, following Karbstein, Prinz, et al. (2020), (v) GD explained by HD; (vi) iFD_CV_ explained by HD, and (vii) iFD_CV_ explained by GD (except for nonsignificant interaction between HD and GD). In total, the model comprises 84 unspecified variable combinations (“independence claims”, assumption about lack of a relationship between variables), of which 10 were significant and classified as correlated errors because they are not causal and/or unidirectional (*N*_A_ ~ CV_altitude_, *H*_e_ ~ *I*, iFD_CV_ ~ CV_slope exposure_, *H*_o_ ~ CV_slope_, *F*_IS_ ~ CV_pH_, population size ~ HD, *I* ~ *N*_A_, *F*_IS_ ~ *H*_o_, and *G*_ST_ ~ *I*) within the SEM structure. To perform the SEM, we applied the function psem within the R package “piecewiseSEM” vers. 2.3.0 (Lefcheck, 2016, 2022).

## 3 RESULTS

### 3.1 Environment, traits, and population size

Central German locations are spread across different landscapes, representing an environmental gradient from relatively warm and dry lowlands (Thuringian Basin) to cooler and wetter Central German mountain systems (Thuringian Forest, Rhoen Mountains; Table 2). Annual temperature (*T*_a_) and annual precipitation (*P*_a_) ranged from 6.7–8.7°C and 511–767 mm, respectively. Riezlern, located in the Northern Alps, was characterized by remarkably cooler *T*_a_ of 5.7°C and higher *P*_a_ of 1045 mm. Habitats are moderately species‐rich (19–33 species in 20 m^2^ transect) and are dominated by a few grass species with minor abundances of other semi‐dry grassland species (E 0.60–0.83). Most *T. montanum* populations grew on north‐ to west‐exposed grassland locations, which varied considerably in density and height of vegetation and thus in biotic competition (LAI 1.2–5.8, crop height 11–83 cm, wL 6.7–7.9, herb layer 76%–99%, bare ground 0%–10%). In addition, habitats were predominantly characterized by flat slopes (2.0–16.1°), slight continentality (wK 3.0–4.5), and shallow (soil depth 9.6–21.5 cm), slightly acidic to calcareous (pH autocorrelated to previously excluded CaCO_3_; pH 5.4–8.0, wR 6.8–7.9), relatively dry (wF 3.1–5.5), and low to medium nutrient‐rich (CEC_pot_ 12.9–17.5 cmol/kg, N 0.36%–0.83%, P 0.6%–2.6%, wN 2.7%–4.6%, K 3.6%–28%) soils.

**FIGURE 2:**
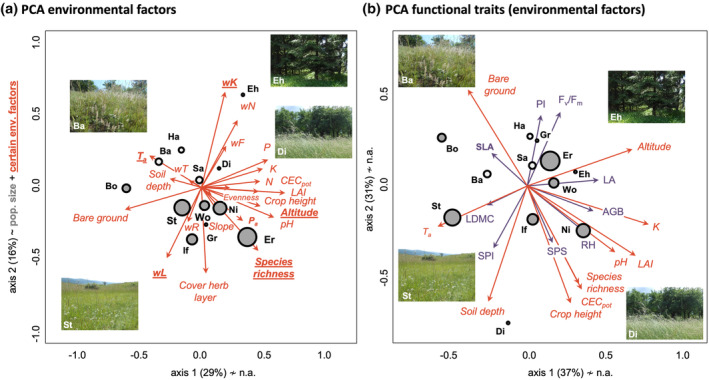
(a) PCA of abiotic and biotic environmental factors based on 12 small and large *Trifolium montanum* populations in Central Germany. Riezlern (KW) was removed from the PCA due to its substantially different climatic conditions compared to the Central German locations, and the ordinal variable slope exposure was excluded from the analysis. The first two principal components explained 45% of variation. PCA axis 2 with correlated factors in bold (wK, wL, *P*_a_, species richness, *T*_a_, altitude) is mainly and significantly responsible for the separation of populations according to size (see Section 3.1). (b) PCA of mean functional traits including 12 *T. montanum* populations in Central Germany. Riezlern (KW) was excluded due to missing abiotic and biotic environmental factors (F_v_/F_m_, PI, and crop height). Here, no PCA axis correlated significantly with population size. Environmental factors significantly correlated with PCA axes are indicated by red arrows. The first two principal components explained 68% of trait variation. The size of location circles represents population size (Table 1). Classification of population size was done at the median of the distribution. Figure inlets show different habitats of *T. montanum*: Ba, Bad Frankenhausen (Ba) – continental dry grasslands; Eh, Ehrenberg – montane meadows; St, Steinthaleben – semi‐dry grasslands; and Di, Dielsdorf – nutrient‐rich meadows. Abbreviations of locations, functional traits, and environmental factors are explained in Tables 1, 2, 3, 4, respectively.

The PCA based on abiotic and biotic environmental factors explained 45% of variation with the first two principal components (Figure 2a). We did not observe clear differentiation between large and small *T. montanum* populations, but large populations are grouped relatively close to each other in the center of the PCA (niche optimum), while small populations tend to be found above the center towards the edge (niche pessimum). PCA axis 2 is mainly responsible for the separation according to population size, as confirmed by significant correlations of PCA2 with population size (*r*_Sp_ = −.70; all other PCA axes *p* > .05), and PCA2 with environmental factors (wK, *r*_Sp_ = .72; wL, *r*_SP_ = −.71; *P*_a_, *r*_SP_ = −.58; SR, *r*_Sp_ = −.58; *T*_a_, *r*_SP_ = .53; altitude, *r*_Sp_ = −.52). Consequently, large compared to small populations tend to be located in extensively used, species‐rich *Bromus erectus* habitats in hilly regions characterized by lower continentality, higher light availability (wL) and annual precipitation (*P*_a_), and lower annual temperatures (*T*_a_). The remaining environmental factors do not differentiate between large and small populations (e.g., soil depth, CEC_pot_). In poorly managed meadows dominated by the grasses *Brachypodium pinnatum* and *Dactylis glomerata* (e.g., Di and Eh; Figure 2a) population sizes are smallest, which is associated with high nutrient availability and biotic competition (high wN, wF, and LAI, low wR). Moreover, all abiotic and biotic environmental factors differed significantly among locations (*p* < .05; Table 2). Variation in population size (50 to 20,900 individuals; Table 1) is not correlated to abiotic habitat heterogeneity (HD; *R* ^2^ = .09, *p* = .32), and HD did not significantly depend on habitat area (*R* ^2^ = .04, *p* = .49).

In contrast to multivariate PCA and related correlation results, a multiple LM between population size and single environmental factors exhibited no significant relationships (*R* ^2^_adj_ = .00, *F*_2,10_ = 0.33, *p* = .90), even after model simplification. Single LMs for each environmental factor separately also revealed no significant associations (all *p* > .05). Interestingly, the variation (CV) of certain environmental factors nearly completely, and significantly explained population size (*R* ^2^_adj_ = .96, *p* < .01): The higher the variation (explained variation in brackets, respectively) of soil depth (18%), altitude (10%), N (9%), and slope exposure (8%) the lower the population size, and the higher the variation of LAI (19%), K (14%), pH (12%), P (5%), and slope (5%), the larger the population size.

The PCA based on functional traits explained 68% of variation with the first two principal components (Figure 2b). Similar to the previous analysis, we observed no clear differentiation between large and small populations, but large populations are grouped in the center of the PCA (niche optimum) whereas small populations are found around the center towards the edges (niche pessima). Therefore, no PCA axis significantly correlated with population size and environmental factors in a linear manner (*p* > .05). Large populations are predominantly characterized by medium functional trait values. More extreme trait combinations are situated in niche‐pessimum conditions, for example, (i) the small continental dry grassland populations (Ba) exhibits individuals with small height, leaf area, and stomata, and of low‐biomass, which correlate with high annual temperatures and light availability but low precipitation; (ii) the small *Arrhenaterum* grassland population (Di) comprises individuals with large height and stomata size but also stressed (low F_v_/F_m_ and PI), associated with high biotic competition (crop height, LAI), soil depth, and nutrient supply (K); (iii) further small populations (Ha, Sa, Gr, Eh) are characterized by unstressed (high F_v_/F_m_ and PI) individuals with small to medium plant height, biomass, and leaf area, correlated with low biotic competition, medium light availability, and low soil depth and pH. In addition, all functional traits differed significantly among locations (*p* < .05, Table 4). LM results also showed that functional traits were significantly affected by their abiotic environment and that the direction of correlation was highly trait‐dependent and complex across traits (Table S2).

### 3.2 Population genetic indices and population size

**FIGURE 3:**
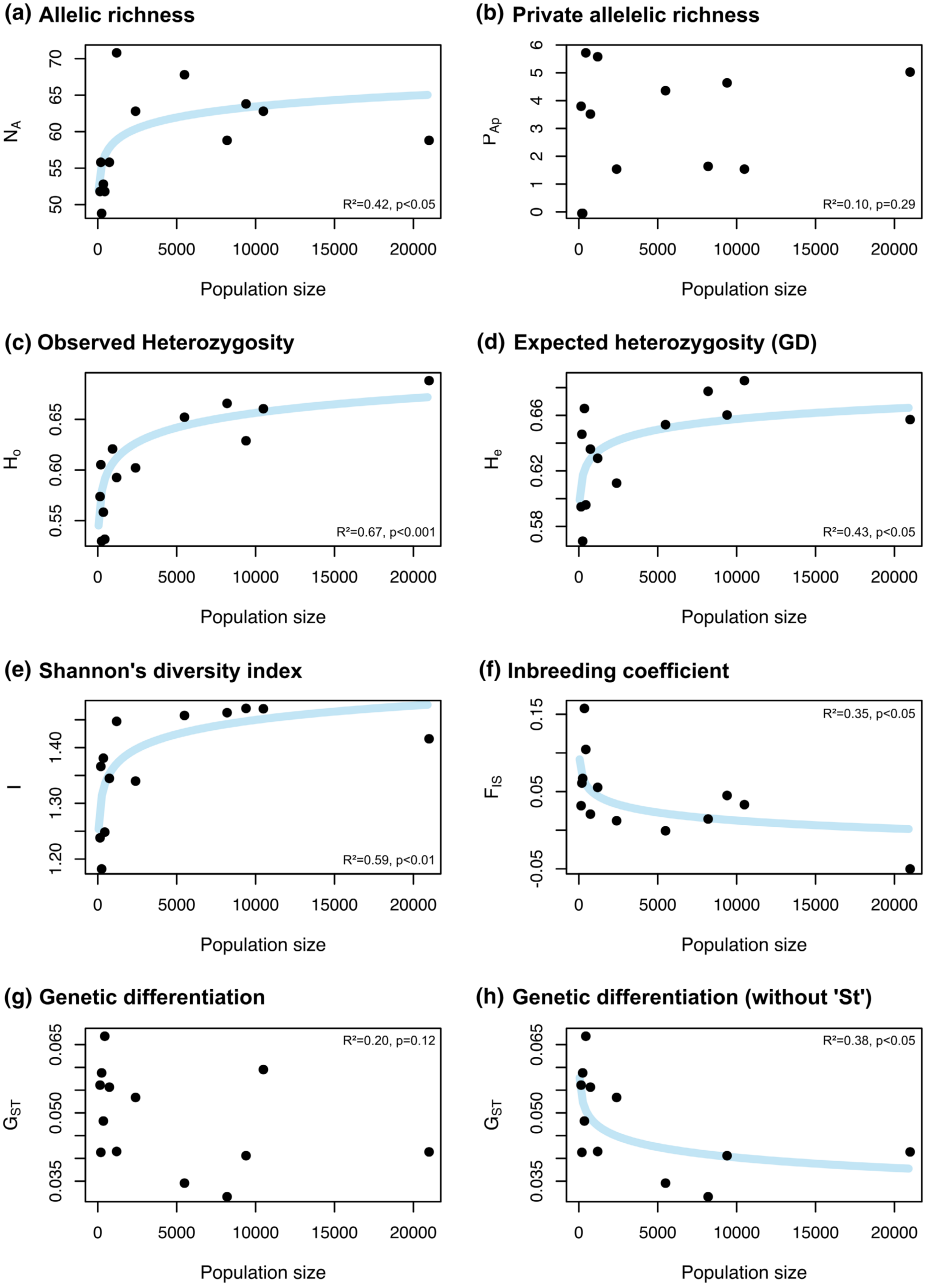
Relationships between population genetic indices (a) allelic richness, (b) private allelic richness, (c) expected heterozygosity, (d) observed heterozygosity, (e) Shannon's diversity index, (f) inbreeding coefficient, (g) genetic differentiation, and (h) genetic differentiation without population St and population size of 13 *Trifolium montanum* populations in Central Europe based on nine microsatellite markers and 255 individuals (see Tables 1 and 4). Linear regression models were performed with log‐transformed population sizes, and *ln*‐functions were fitted to the untransformed data set. Curves of nonsignificant relationships (*p* > .05) were not drawn.

*Trifolium montanum* populations are characterized by a range of allelic richness (*N*_A_) from 49 to 71 alleles, private allelic richness (*P*_Ap_) from 0 to 5.8%, observed heterozygosity (*H*_o_) from 0.531 to 0.690, expected heterozygosity (*H*_e_) from 0.570 to 0.686, Shannon's information index (*I*) from 1.185 to 1.473, inbreeding coefficient (*F*_IS_) from −0.049 to 0.159, and genetic differentiation (*G*_ST_) from 0.032 to 0.067 (Table 3). LMs revealed significant positive mean relationships between population size and genetic diversity indices *N*_A_ (*R* ^2^ = .42, *p* < .05), *H*_o_ (*R* ^2^ = .67, *p* < .001), *H*_e_ (*R* ^2^ = .43, *p* < .05), and *I* (*R* ^2^ = .59, *p* < .01; Figure 3a,c‐e). F_IS_ is significantly negatively related to population size (*R* ^2^ = .35%, *p* < .05; Figure 3f). No significant linear relationships were found between population size and *P*_Ap_ (*R* ^2^ = .10, *p* = .29; Figure 3b) and *G*_ST_ (*R* ^2^ = .20%, *p* = .13; Figure 3g). Population size and *G*_ST_ are significantly negatively associated (*R* ^2^ = .38%, *p* < .05) when the population Steinthaleben (St) is removed (Figure 3h).

Regarding the relationships between population genetic indices, the strongest significant positive relationships were found between *H*_e_ and *I* (*r*_P_ = .90, *p* < .001), followed by *H*_o_ and *I* (*r*_P_ = .79, *p* < .01), *N*_A_ and *I* (*r*_P_ = .79, *p* < .01), *H*_o_ and *H*_e_ (*r*_P_ = .77, *p* < .01), and H_o_ and *N*_A_ (*r*_P_ = .57, *p* < .05). The strongest significant negative relationships were observed for *H*_o_ and *F*_IS_ (*r*_P_ = −.77, *p* < .01), followed by *I* and *G*_ST_ (*r*_P_ = −.70, *p* < .01), *H*_o_ and *G*_ST_ (*r*_P_ = −.61, *p* < .05), and *H*_e_ and *G*_ST_ (*r*_P_ = −.58, *p* < .05). *P*_Ap_ was not significantly associated with other diversity indices (all *p* > .05). For single microsatellite loci, similar relationships between genetic indices and population size were detected (not shown). Allelic size ranges were comparable to Matter et al. (2012), except for locus *Tm21*, which showed a three‐fold larger size range (Table S2). The total *N*_A_ was 146, including all nine microsatellite loci.

### 3.3 Path analysis to model iFD_CV_ (ITV)

**FIGURE 4:**
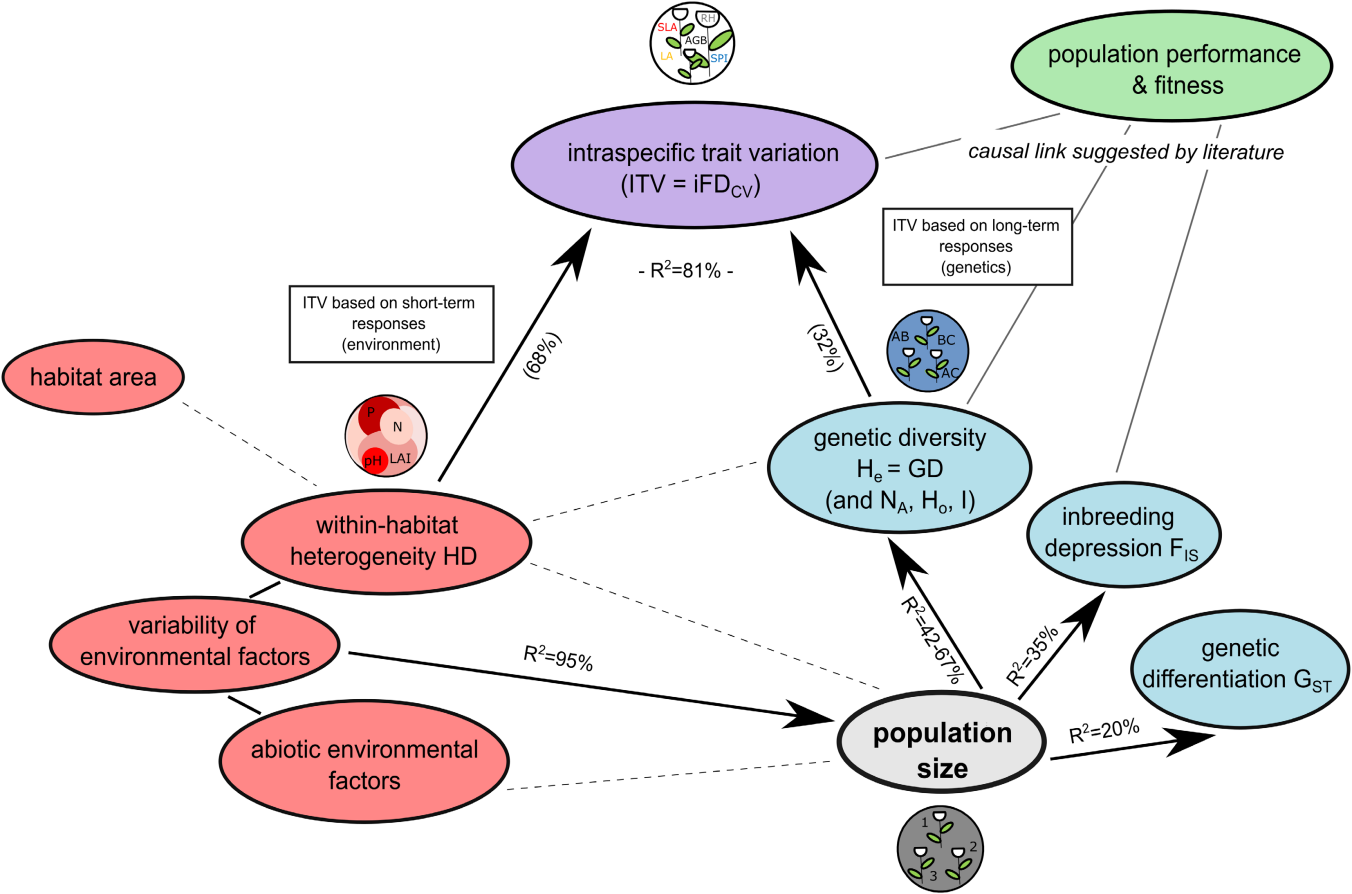
A framework of inferred relationships in *Trifolium montanum* among intraspecific trait variation (iFD_CV_), abiotic within‐habitat heterogeneity (HD), population genetic diversity (*N*_A_, *P*_Ap_, *H*_e_ = GD, *H*_o_, *I*), inbreeding (*F*_IS_), and differentiation indices (*G*_ST_) shaped by population size, which is in turn affected by variation of abiotic environmental habitat factors. Results are based on the SEM analysis (local SEM; see Section 3.3 for details). Significant relationships are indicated with solid black arrows, whereas nonsignificant relationships are shown as dashed lines. Black solid lines indicate dependencies due to mathematical calculations, and gray lines assumed causal relationships from examined literature (link between genetic diversity inbreeding/trait variation to plant population performance and fitness). The color scheme was taken from previous Figures 2 and 3, and the basic concept from Karbstein, Prinz, et al., 2020 (published under Creative Commons License, redrawn herein). *N*_A_ = allelic richness (total number of alleles), *H*_e_ = expected heterozygosity, *H*_o_ = observed heterozygosity, *F*_IS_ = inbreeding coefficient, *I* = Shannon's diversity index, *G*_ST_ = differentiation of a given population relative to all populations.

The path analysis (local SEM) represents the formulated hypothesis and underlying data well (Fisher's *C* = 146.50, *p* = .52; Table S3). We predominantly inferred significant, moderately to highly explained relationships between the response variables population size (*R* ^2^ = .93), HD (*R* ^2^ = .05), *N*_A_ (*R* ^2^ = .42), *H*_o_ (*R* ^2^ = .67), *H*_e_ (*R* ^2^ = .43), *I* (*R* ^2^ = .59), *F*_IS_ (*R* ^2^ = .35), *G*_ST_ (*R* ^2^ = .20), iFD_CV_ (*R* ^2^ = .81), and their respective predictors (Figure 4). Effect direction of variables and explained variation of relationships is similar to previous analysis here and in Karbstein, Prinz, et al. (2020).

In detail, we observed significant relationships between population size and CV_LAI_ (23%, *p* < .01), CV_soil depth_ (22%, *p* < .01), CV_K_ (21%, *p* < .01), CV_N_ (12%, *p* < .05), CV_pH_ (12%, *p* < .05), and CV_altitude_ (10%, *p* < .05). In contrast to previous LM analyses, CV_P_ and CV_slope exposure_ were not correlated with population size. With increasing variation in soil depth, soil nitrogen content, and altitudes, and decreasing variation of light availability, soil potassium content, and soil pH within habitats, population sizes become larger. Variation of habitat environmental factors (HD) is not statistically affected by habitat area (*p* = .49), as also shown previously. All population genetic diversity indices are again significantly positively affected by population size, with strongest relationships found for *H*_o_ (*p* < .001), followed by *I* (*p* < .01), *N*_A_ (*p* < .05), and *H*_e_ (GD; *p* < .05). With increasing population size, inbreeding (*F*_IS_) decreased significantly (*p* < .05), and genetic differentiation (*G*_ST_) again showed no significant association with population size (*p* = .12; *G*_ST_ without the population ST would have led to the entire exclusion of this population in path analyses). HD did not statistically significantly explain *H*_e_ (GD; *p* = .24). Intraspecific trait variation (iFD_CV_) is significantly positively explained by both HD (68%, *p* < .001) and GD (32%, *p* < .05).

## 4 DISCUSSION

Understanding the characteristics and responses of plant populations under different environmental conditions is an important evolutionary and ecological challenge, and a critical target of biodiversity and nature conservation research. This study unraveled population size as an important prerequisite of population performance derived from plant functional traits; it shed new light on complex, population size‐dependent genotype–phenotype‐environment interactions by recording detailed location‐wise information about abiotic and biotic environmental habitat factors, genetic diversity, inbreeding and differentiation, and functional traits (ITV) using up‐to‐date statistical modeling approaches within a multivariate framework (Figure 4).

In particular, our study made progress by comprehensively reconstructing relevant processes that influence ITV and population performance, and unraveling population size as the most critical factor. In contrast to expectations, population size was not linearly affected by abiotic environmental factors (see also center vs. niche distribution, Figure 2a) but was almost completely explained by the variation of certain abiotic environmental habitat factors. With rising population size, genetic diversity (*H*_e_, *N*_A_, *H*_o_, *I*) increased, whereas inbreeding (*F*_IS_) and genetic differentiation (*G*_ST_) decreased in *T. montanum* (Figure 3). Finally, ITV (iFD_CV_) could be largely attributed to habitat heterogeneity (68%) and to a lesser extent to genetic diversity (*H*_e_, 32%; Figure 4). Population size via population genetic consequences, therefore, represents an important, but interestingly not the most important factor shaping ITV in *T. montanum* populations. The here investigated positive relationships among population size, genetic diversity, and ITV as an indicator for performance (e.g., Hensen et al., 2005; Leimu et al., 2006; Reisch et al., 2021; Rosche et al., 2022), and among genetic diversity, habitat heterogeneity, and ITV (e.g., Karbstein, Prinz, et al., 2020; Waitt & Levin, 1998) are consistent with literature. In general, small as opposed to large *T. montanum* populations are characterized by medium to extreme environmental habitat factor and functional trait values (several niche pessima), higher (LAI, soil K and pH) and lower (soil depth and N, altitude) variability of certain abiotic environmental factors, lowered genetic diversity, elevated inbreeding and differentiation, and finally lower ITV and performance (Table 5).

**TABLE 5:** Characterization of large and small *T. montanum* populations in terms of environment, genetics, and traits and according to the results of this study (Figures 2, 3, 4, Tables 1, 2, 3, 4, see Section 2.3 for population classification).

Factor/index/trait	Large populations	Small populations
Abiotic and biotic environment (PCA)	Center distribution (medium environmental factor values)	Center‐to‐edge distribution (medium to extreme environmental factor values)
Functional traits (PCA)	Center distribution (medium functional trait values)	Edge distribution (towards different extreme functional trait value combinations)
Single habitat factors	– (n.s.)	– (n.s.)
Variation (CV) of single habitat factors	↑ (CV_soil depth_, CV_N_, CV_altitude_), ↓ (CV_LAI_, CV_K_, CV_pH_)	↑ (CV_LAI_, CV_K_, CV_pH_), ↓ (CV_soil depth_, CV_N_, CV_altitude_)
Within‐habitat heterogeneity (HD)	‐ (n.s.)	‐ (n.s.)
Genetic diversity	↑ (*N*_A_, *H*_o_, *H*_e_ = GD, *I*)	↓ (*N*_A_, *H*_o_, *H*_e_ = GD, *I*)
Genetic inbreeding	↓ (*F*_IS_)	↑ (*F*_IS_)
Genetic differentiation	↓ (*G*_ST_)	↑ (*G*_ST_)
Intraspecific trait variation (ITV)	↑ (iFD_CV_)	↓ (iFD_CV_)

Abbreviations: − (n.s.), no significant relationship found; ↑, increased; ↓, decreased; *F*_IS_, inbreeding coefficient; *G*_ST_, differentiation of a population relative to all populations; *H*_e_, expected heterozygosity; *H*_o_, observed heterozygosity; *I*, Shannon's diversity index; *K*, soil potassium content; *N*, soil nitrogen content; *N*_A_, allelic richness; *P*_Ap_, private allelic richness.

### 4.1 Environment, traits, and population size

The results show no clear differentiation of environmental factors or functional traits between small and large *T. montanum* populations but revealed that large populations are characterized by intermediate values (center distribution), while small populations showed intermediate to extreme value combinations (center‐to‐edge distribution; Figure 2a,b, Table 5). This observation is consistent with ACH predictions and observations for *T. montanum* that optimal habitat quality (niche optimum) leads to high growth rates and population sizes, whereas nonoptimal habitat quality (niche pessimum) results in low growth rates and small population sizes, which are characterized by nontypical or novel phenotypic trait responses (Brown, 1984; Hirsch et al., 2015; Leimu et al., 2006; Schleuning et al., 2009; Schleuning & Matthies, 2008). Large *T. montanum* populations were more abundant in extensively used, species‐rich *Bromus erectus* (*Mesobromion*) habitats with relatively high light availability, moderate continentality, temperatures, and precipitation, and low biotic competition, located along shrub and forest edges as well as way‐ and roadsides. According to the literature, *T. montanum* should occur predominantly in nutrient‐poor, calcareous, sub‐Mediterranean to continental grasslands (Jäger, 2011; Schleuning et al., 2009), but we have also found populations of various sizes on weak acidic to pH‐neutral (e.g., in Bottendorf Bo, Bad Frankenhausen Ba), moderately nutrient‐rich soils (e.g., in Niederwillingen Ni, Steinthaleben St), suggesting tolerance to different pH and nutrient conditions in Central Germany.

Although *T. montanum*'s range center is situated in Eastern Europe characterized by rather continental climate, the species prefers moderate *Mesobromion* meadows in Central Germany and did not cope well with continental steppe grasslands (e.g., Bo, Ba). These continental grassland populations comprise individuals with low plant heights (RH) and biomass (AGB), smaller stomata (SPS), potential stomatal conductance (SPI), and small (LA), more robust leaves (LDMC). Individuals at these locations respond to increased heat, drought, and light stress. This is known to affect nutrient uptake, photosynthesis, plant growth, and thus certain functional traits (Bucher et al., 2017; Cornelissen et al., 2003; Farooq et al., 2009; Jaleel et al., 2009; Karbstein et al., 2019; Pérez‐Harguindeguy et al., 2013). Another niche pessimum with small populations is situated in poorly managed lowland and montane meadows (e.g., Ehrenberg Eh, Dielsdorf Di) mainly consisting of *Arrhenaterum elatius*, *Dactylis glomerata*, and *Medicago falcata*. These locations exhibit nutrient‐rich, pH‐neutral, humid soils, and dense vegetation with high biotic competition. To deal with these conditions, *T. montanum* individuals probably become larger (with medium biomass and leaf size, large stomata, and high stomatal conductance) to compete for light with surrounding grass species (Cornelissen et al., 2003; Gaudet & Keddy, 1988; Moles et al., 2009). Individuals also show increased stress (F_v_/F_m_) and reduced vitality (PI; Björkman & Demmig, 1987; Clark et al., 2000; Johnson et al., 1993; Kalaji et al., 2012; Maxwell & Johnson, 2000; Strasser et al., 2000), which indicates reduced performance. In accordance with results presented here, other *T. montanum* studies observed that light competition decreases survival probability of juvenile plants particularly in unmanaged sites, resulting in aged and smaller populations (Schleuning et al., 2009; Schleuning & Matthies, 2008).

### 4.2 Environment, population size, genetics, and ITV

Population size is almost entirely explained by abiotic environmental variation within habitats. Although many studies have examined relationships among plant population size, genetic diversity, and/or performance or fitness (e.g., De Kort et al., 2021; Leimu et al., 2006; Rosche et al., 2022; Szczecińska et al., 2016), they have focused less on how population growth rate and size depend on environmental habitat factors and/or variation within these statistical frameworks (Lawson et al., 2015; Nicolè et al., 2011; Schleuning et al., 2009; Schleuning & Matthies, 2008). Plants are sessile organisms, and thus particularly susceptible and vulnerable to spatiotemporal environmental variation (Karbstein et al., 2019; Nicolè et al., 2011). Environmental factors thus likely influence population growth rates and size in *T. montanum* (e.g., as shown for light intensity and biotic competition in Schleuning et al., 2009; Schleuning & Matthies, 2008), but *T. montanum* is a less competitive semi‐dry grassland species, requiring extensive grassland land‐use management to ensure long‐term viability of populations. For example, large *T. montanum* populations are characterized by increased LAI, soil pH, K, and slope (Figure 4). Increased variation in these factors indicates habitats with patches of high and low light, specific nutrients, and biotic competition conditions that reduce the dominance of grass species and allow the presence of less competitive species like *T. montanum*. In contrast, reduced variation in slope exposure, slope, and soil N leads to large population sizes because *T. montanum* prefers north‐exposed, flat, rather nutrient‐poor habitats. Spatial variation in environmental factors thus overrides the effects of mean environmental factors, a phenomenon that has rarely been studied in detail in plant populations (temporal variation reviewed, e.g., in Lawson et al., 2015).

In *T. montanum*, population size strongly determines genetic diversity (*N*_A_, *H*_o_, *H*_e_, *I*) and inbreeding (*F*_IS_), and partly differentiation (*G*_ST_, Figures 3 and 4). Large *T. montanum* populations such as ST or Er with more than 10,000 individuals show increased genetic diversity and decreased inbreeding, high ITV, and good performance (as directly indicated by PI and F_v_/F_m_; Table 4). These large populations probably contain many different, heterozygous genotypes due to increased gene flow (efficient pollinator activity in large populations) and genetic recombination, and thus less inbreeding and genetic drift effects. In contrast, very small *T. montanum* populations such as Di surrounded by agrarian areas (Figure S2) are likely to suffer under restricted gene flow within but also with surrounding populations, and thus perpetuating and amplifying genetic drift, inbreeding, and decreasing performance (as directly indicated by PI and F_v_/F_m_; Table 4) may result in extinction  (Freeland et al., 2011; Leimu et al., 2006; Rosche et al., 2022; Schleuning et al., 2009).

In general, associations between population genetic indices and population size are strong at low and weak or absent (“saturated”) at larger sizes (reviewed in Leimu et al., 2006; e.g., Luijten et al., 2000; Rosche et al., 2022). This general pattern was also confirmed here, except for private allelic richness probably due to the geographically narrow sampling (Kalinowski, 2004; Figure 3). In contrast to Leimu et al. (2006), in small *T. montanum* populations, the loss of allelic richness and thus genetic drift was less important compared to the loss of observed heterozygosity and thus homozygosity and inbreeding. The long‐lived nature of mountain clover (Figure S1) might explain this observation, as perennials compared to annuals are less vulnerable to pollinator limitation or demographic stochasticity in recruitment, and genetic drift (Freeland et al., 2011; Hamrick et al., 1979; Leimu et al., 2006).

Interestingly, Leimu et al. (2006) investigated no general relationship between inbreeding and population size across species due to equivocal results between self‐compatible and self‐incompatible species. In *T. montanum*, reduced genetic diversity at self‐incompatibility loci probably leads to a decreased number of potential mating partners in populations and a decrease in female fitness (Fischer et al., 2003; Karbstein, Rahmsdorf, et al., 2020; Willi et al., 2005), which is supported by observation of strong decrease in reproduction with dropping local individual density and pollinator activity (Schleuning et al., 2009). Consequently, with decreasing size, homozygotes meet more frequently, accelerating the vortex of inbreeding and extinction. A breakdown in self‐incompatibility may alter these relationships (e.g., Porcher & Lande, 2005; *Trifolium*: Frye & Neel, 2017), but this has not yet been observed in *T. montanum*.

Self‐incompatible species, as already mentioned, but also neutral DNA markers like microsatellites favor a strong relationship between population size and genetic diversity (Leimu et al., 2006). Natural selection is less acting on neutral markers leading to higher genetic variability and thus increased potential in explaining relationships to population size (Frankham, 1996; Leimu et al., 2006; Rosche et al., 2022). Genetic differentiation is also elevated in smaller *T. montanum* populations. This is probably caused by less gene flow with surrounding populations due to habitat fragmentation (e.g., small population Dielsdorf surrounded by agrarian area, and Ehrenberg surrounded by forests, Figure S2) and density‐dependent pollinator activity within populations, leading to higher genetic isolation of smaller populations.

Genetic diversity (*H*_e_) represents a critical prerequisite for high variability of functional traits within populations, ITV (iFD_CV_), and therefore population performance (Figure 4). Nevertheless, ITV in *T. montanum* populations is mainly generated by the response of genotypes to abiotic environmental habitat heterogeneity (HD). The interaction between *H*_e_ and HD did not affect ITV, suggesting phenotypic plasticity‐based ITV, rather than ITV associated with specific site‐adapted genotypes. Accordingly, although abiotic environment can act on genetic diversity via natural selection (Linhardt & Grant, 1996; Reisch et al., 2021; Sakaguchi et al., 2019), we did not detect selective pressure here, likely due to the applied neutral marker type and to insufficient abiotic selective pressures within habitats (Figure 4). Increased ITV based on functional traits (i.e., number of different functional phenotypes, e.g., small‐high plants with low‐high biomass tender‐robust leaves, and low‐high stomatal conductance) and healthy individuals (i.e., moderate to high photosynthetic performance and stress indicators) shows that many large populations perform well under given environmental conditions and concerning their genetic background, implying positive plant performance. Taken together, these observations also fit ACH predictions that populations decline in size and genetic and phenotypic diversity due to decreasing habitat quality and increasing negative effects of inbreeding, genetic drift, restricted gene flow as well as elevated genetic differentiation (Brown, 1984; Hampe & Petit, 2005; Hardie & Hutchings, 2010; Hirsch et al., 2015; Hoffmann & Blows, 1994; Sagarin et al., 2006; Wagner et al., 2012).

### 4.3 Long‐term viability and nature conservation of *Trifolium montanum* populations in semi‐dry grasslands

This research improves the theoretical understanding of relationships among population size, environment, genetic diversity, and inbreeding, and ITV as an indicator of plant performance. It has several implications for applied biodiversity and nature conservation. *Trifolium montanum* populations in nonoptimal habitats are characterized by reduced genetic and intraspecific functional trait diversity, and increased genetic inbreeding and differentiation. These signals indicate a decreased plant performance and fitness, and therefore, reduced adaptability to current and future environmental changes, and elevated extinction risk (Ellstrand & Elam, 1993; Karbstein, Rahmsdorf, et al., 2020; Leimu et al., 2006; Spielman et al., 2004). The fate and long‐term survival of small populations will be highly dependent on adequate habitat protection and land‐use actions to stabilize population sizes and escape the vortex of extinction (Ellstrand & Elam, 1993; Leimu et al., 2006; Rosche et al., 2022). For example, habitat degradation and fragmentation are well‐known to reduce population size and density, increase isolation, and limit gene flow, all of which negatively affect genetic diversity and ITV (Hensen et al., 2005; Hensen & Wesche, 2006; González et al., 2020; Karbstein, Prinz, et al., 2020). In order to stabilize or rescue small *T. montanum* populations, it is important to first improve habitat quality according to environmental preferences (niche optimum) to ensure sufficiently high population growth rates, and second, to increase the habitat area of a given population, either by enlarging suitable habitat area or by connecting previously isolated habitats. Applied to *T. montanum* populations in Central Germany, optimal habitats are characterized by extensively managed, species‐rich, calcareous *Bromus erectus* semi‐dry grasslands with low vegetation density (less biotic grass competition), and moderate soil nutrient supply and humidity (Figure 2a).

For *T. montanum*, studies have shown that the consequences of habitat degradation are more important than those of habitat fragmentation in the short term. In unmanaged sites, population growth rates decrease with increasing light competition (LAI) because of higher investment in plant height and lower investment in flowering structures, recruitment, and survival, resulting in aged populations (Schleuning et al., 2009; Schleuning & Matthies, 2008). Extinction in these perennials is likely to take a long time, and even very small populations can persist for decades until extinction (e.g., up to ca. 30 years old individual observed in this study, Figure S1b). Currently, abandonment of land use and habitat eutrophication due to nitrogen deposition are most problematic for open, oligotrophic grasslands, allowing for the dominance of certain grasses while reducing less competitive species (Habel et al., 2013) such as *T. montanum*. Appropriate land‐use management (e.g., frequent animal grazing, or occasional mowing to prevent succession) can rapidly increase population growth rates of even small *T. montanum* populations and reduce the risk of population extinction (Schleuning et al., 2009). Small populations revealed relatively low but still moderate genetic diversity and signs of inbreeding, suggesting that populations may have a temporally limited potential to persist in these nonoptimal habitats. Interestingly, individuals from these small *T. montanum* populations were often not highly stressed (Figure 2b, Tables 2, 3, 4). Good nutrient and water supply, and moderate biotic competition and inbreeding may explain this observation. Nevertheless, in general, adequate nature conservation actions need to be taken in the near future to ensure the long‐term survival of *T. montanum* populations.

## AUTHOR CONTRIBUTIONS

**Kevin Karbstein:** Conceptualization (equal); data curation (lead); formal analysis (equal); funding acquisition (supporting); investigation (lead); methodology (equal); project administration (supporting); resources (supporting); software (lead); supervision (supporting); validation (lead); visualization (lead); writing – original draft (lead); writing – review and editing (lead). **Christine Römermann:** Conceptualization (lead); data curation (supporting); formal analysis (supporting); funding acquisition (lead); investigation (supporting); methodology (equal); project administration (lead); resources (lead); software (supporting); supervision (lead); validation (supporting); visualization (supporting); writing – original draft (supporting); writing – review and editing (equal). **Frank Hellwig:** Conceptualization (lead); data curation (supporting); formal analysis (supporting); funding acquisition (lead); investigation (supporting); methodology (supporting); project administration (equal); resources (lead); software (supporting); supervision (equal); validation (supporting); visualization (supporting); writing – original draft (supporting); writing – review and editing (supporting). **Kathleen Prinz:** Conceptualization (lead); data curation (equal); formal analysis (equal); funding acquisition (lead); investigation (supporting); methodology (equal); project administration (lead); resources (lead); software (equal); supervision (supporting); validation (equal); visualization (supporting); writing – original draft (supporting); writing – review and editing (equal).

## CONFLICT OF INTEREST STATEMENT

None.

## CODE AVAILABILITY

R scripts used in analyses are available from the corresponding author on request.

## Supporting information


Appendix S1

## Data Availability

Basic data supporting the findings of this study are available within the manuscript and the Appendix S1. Environmental, genetic, and functional trait data are available on Dryad data repository (https://doi.org/10.5061/dryad.n02v6wwtd). Functional trait data are additionally deposited on TRY database (www.try‐db.org).
