# Correction to population size affected by environmental variability impacts genetics, traits, and plant performance in *Trifolium montanum* L.

**DOI:** 10.1002/ece3.10563

**Published:** 2023-09-20

Karbstein, K., Römermann, C., Hellwig, F., & Prinz, K. (2023). Population size affected by environmental variability impacts genetics, traits, and plant performance in *Trifolium montanum* L. *Ecology and Evolution*, **13**, e10376. https://doi.org/10.1002/ece3.10376

We apologize for the following errors:

‐ In the Introduction, the reference “Karbstein, Prinz, et al., 2020” must be replaced with “Karbstein et al., 2019,”: “However, highly genetically differentiated, niche‐pessimum/marginal range populations may still have sufficient genetic variation and can be valuable sources and important targets for nature conservation efforts due to site‐specific adaptations (Karbstein et al., 2019; Kirschner et al., 2020).”

In the Material & Methods, “pollinator failure” needs to be replaced with “pollination failure”: “the mean number of seeds per fruit head varies considerably between populations, probably due to pollination failure in small populations.”

‐ In Figure 3, the captions of (b) and (d) contained typos (e.g., “allelelic”), and thus Figure 3 needs to be replaced by the newly supplied Figure 3.

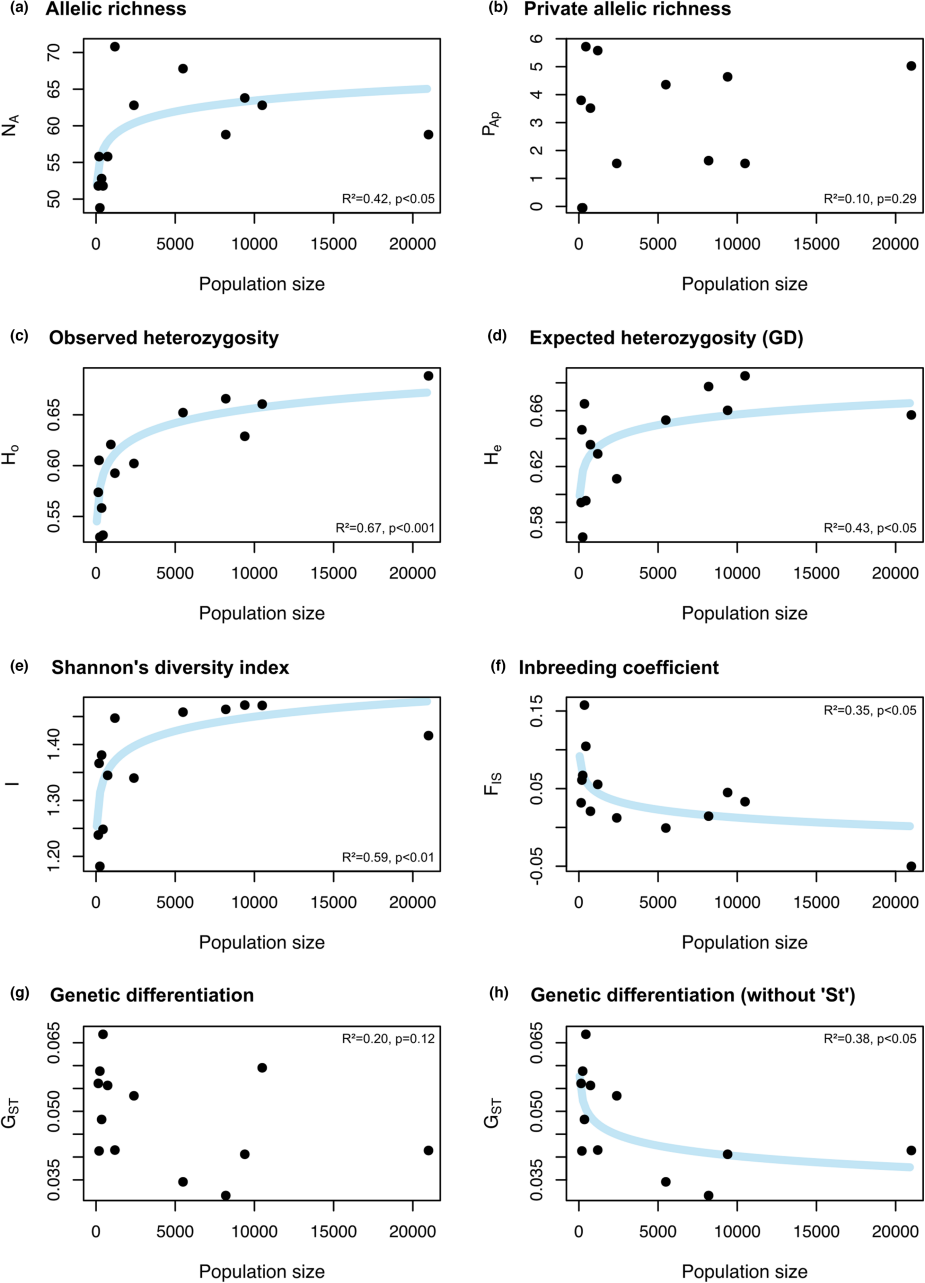

‐ In the Results, the term “transect” must be removed, as the 20 m^2^ refers to the five plots recorded in each location: “Habitats are moderately species‐rich (19–33 species in 20 m ^2^).”

‐ In the Results, the effect direction of certain relationships was mixed up, so please replace the specific text part with “The higher the variation (explained variation in brackets, respectively) of soil depth (18%), altitude (10%), N (9%), and slope exposure (8%) the larger the population size, and the higher the variation of LAI (19%), K (14%), pH (12%), P (5%), and slope (5%), the lower the population size.”

‐ In the Discussion section, the text part concerning environmental habitat variation in relation to population size needs to be replaced because the direction of effects was mixed up while interpreting the results.

Old text: “For example, large *T*. *montanum* populations are characterized by increased LAI, soil pH, K, and slope (Figure 4). Increased variation in these factors indicates habitats with patches of high and low light, specific nutrients, and biotic competition conditions that reduce the dominance of grass species and allow the presence of less competitive species like *T*. *montanum*. In contrast, reduced variation in slope exposure, slope, and soil N leads to large population sizes because *T*. *montanum* prefers north‐exposed, flat, rather nutrient‐poor habitats.”

New text: “For example, increased variation in soil depth and nitrogen, and altitudinal variation (Table 5) leads to large populations because different nutrient conditions on shallow to deep soils in hilly habitats reduce the dominance of grass species and enable the existence of less competitive species like *T. montanum*. Large populations of this species are also characterized by reduced variation (but relatively high values, Figure 2a) of LAI and soil pH, indicating the preference of *T. montanum* to grow under low light and biotic competition on rather calcareous soils.”

