# Supplementary material for: Population size affected by environmental variability impacts genetics, traits, and plant performance in Trifolium montanum L
Source: Ecol Evol. 2023 Aug 7;13(8):e10376. doi: 10.1002/ece3.10376 (PMC10406824; doi:10.1002/ece3.10376)
Supplement: Supplementary file 1 — Appendix S1 [file ECE3-13-e10376-s001.docx]

**Supporting Information**

**Population size affected by environmental variability impacts genetics, traits, and plant performance in *Trifolium montanum* L.**

**Karbstein, Kevin^1,3,4*^, Römermann, Christine^1,2^, Hellwig, Frank^1^, Prinz, Kathleen^1^**

1. *Institute of Ecology and Evolution, Friedrich-Schiller-University Jena, Jena, Germany*
2. *German Centre for Integrative Biodiversity Research (iDiv) Halle-Jena-Leipzig, Germany*
3. *Department of Systematics, Biodiversity and Evolution of Plants (with Herbarium), Albrecht-von-Haller Institute for Plant Sciences, University of Göttingen, Göttingen, Germany*
4. *Max Planck Institute for Biogeochemistry, Department of Biogeochemical Integration, Jena, Germany*

* **Corresponding author:** kkarb@bgc-jena.mpg.de

(a)


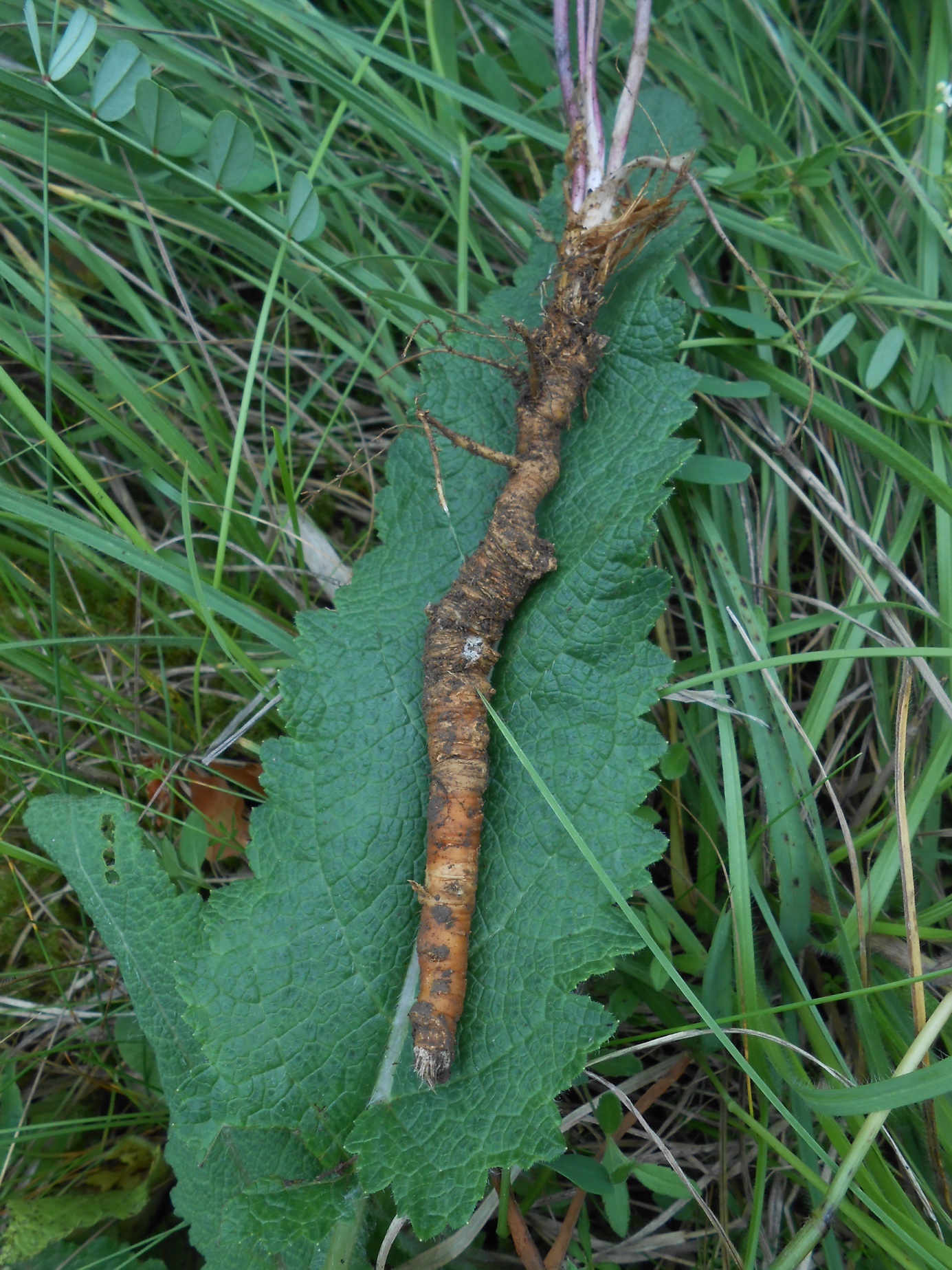


(b)


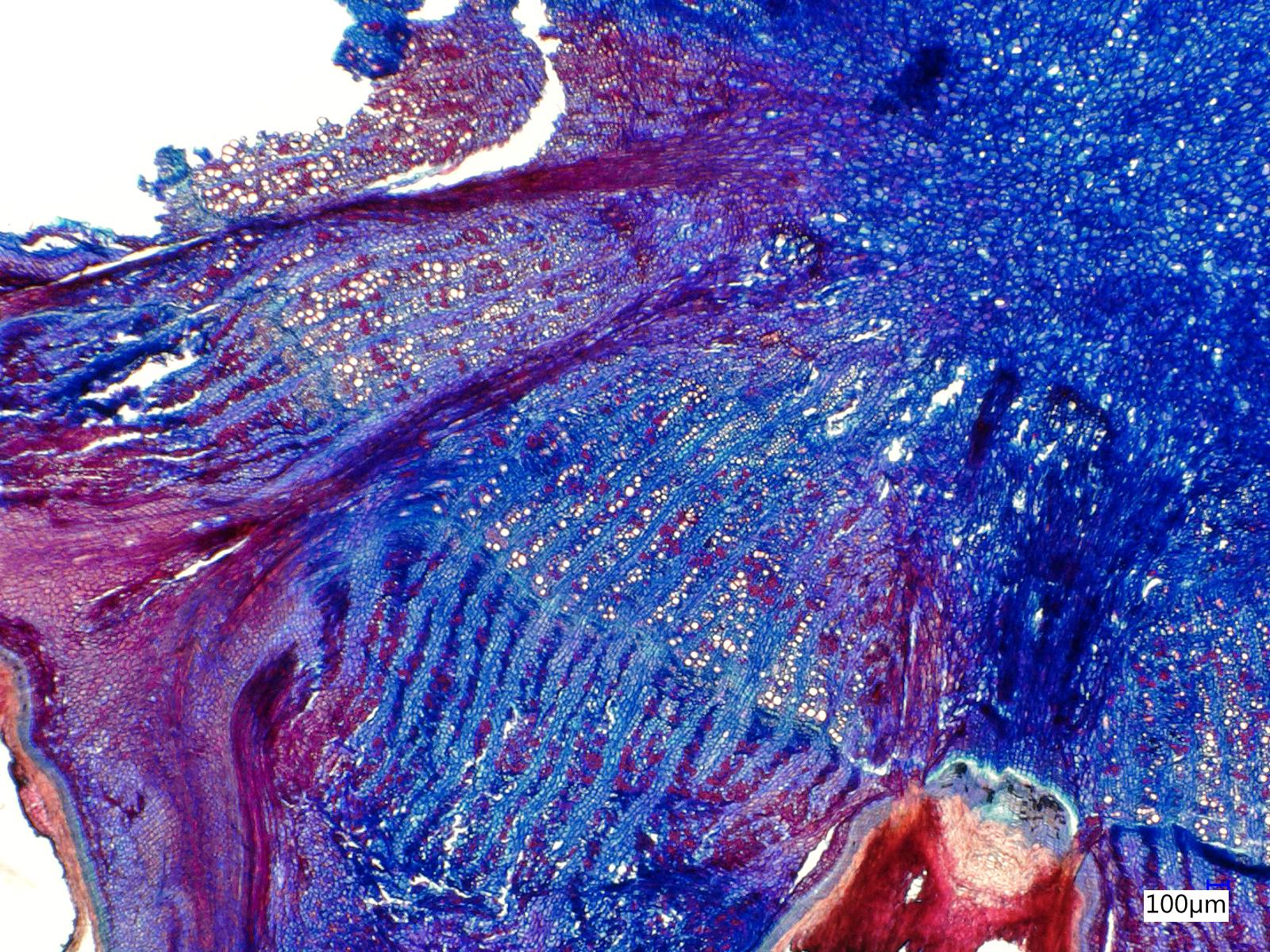


**Fig. S1.** (a) Tap root of an individual from location Niederwillingen (Ni; Table 1), which is more than 20 cm long. (b) Cross-section of a tap root of an individual from location Jena-Wogau (Wo, Table 1), which shows annual growth ring structures. The image was recorded with a VHX-5000 digital microscope (Keyence Deutschland GmbH, Neu-Isenburg, Germany). The cross-section was made by Janin Naumann, and about 30 annual growth rings were counted.


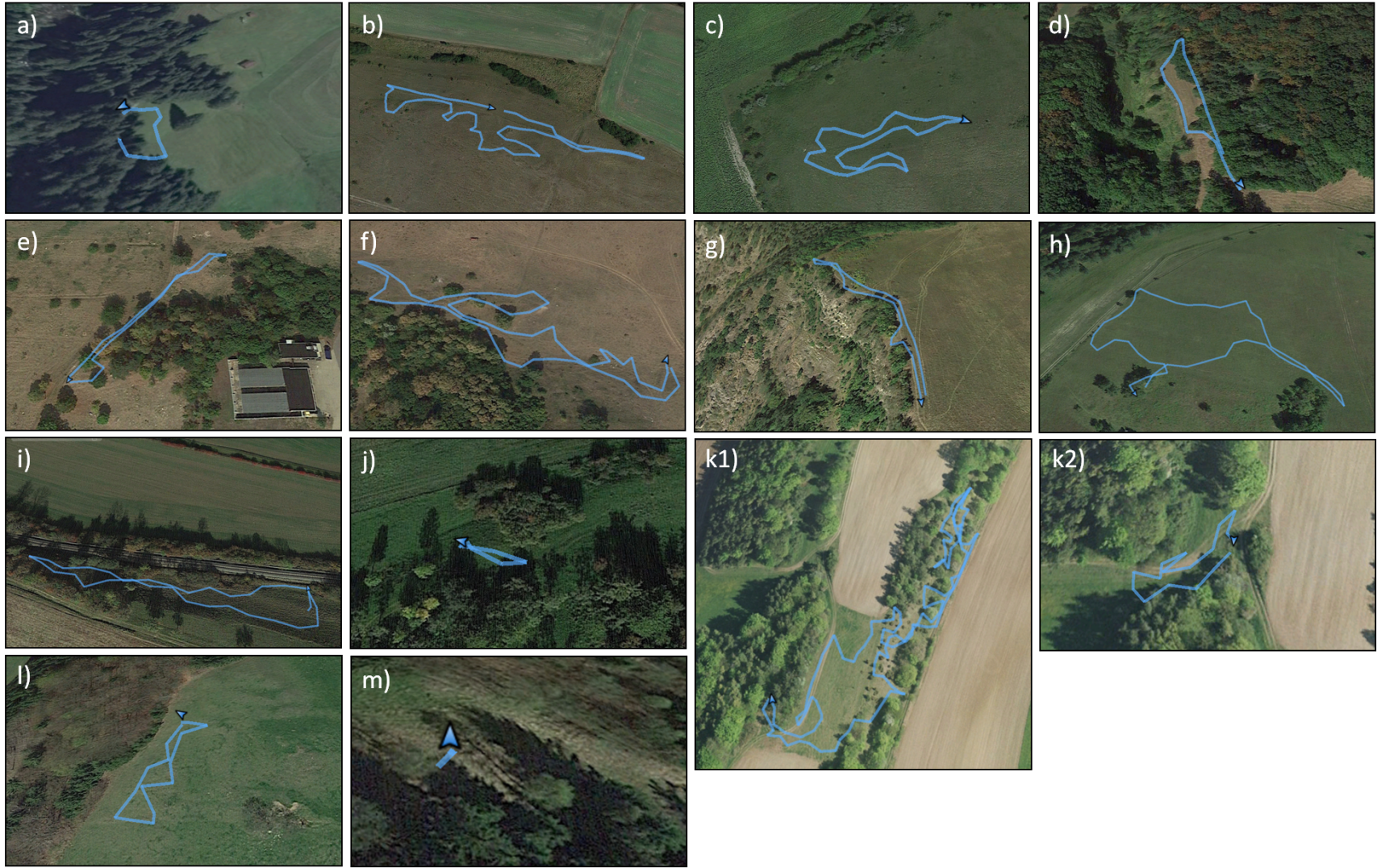


**Fig. S2.** GPX-tracks of 13 T. montanum populations in Central Europe. a) Riezlern (KW, ‘Kleinwalsertal’), b) Bottendorf (Bo, ‘Bottendorfer Hügel’), c) Hardisleben (Ha, ‘Löbehügel’), d) Jena-Wogau (Wo, ‘Hirschberg’), e) Bad-Frankenhausen (Ba, ‘Schlachtberg’), f) Steinthaleben (St, ‘Badraer Schweiz’), g) Saalfeld (Sa, ‘Am Bohlen’), h) Ifta (If, ‘Ziegental’), i) Niederwillingen (Ni, ‘Am Bahndamm’), j) Dielsdorf (Di, ‘Artberg’), k1-2) Erbenhausen (Er, ‘Struthberg’, two subpopulations), l) Großneundorf (Gr, ‘Hainkuppe’), and m) Ehrenberg (Eh, ‘Kappellenberg’). Figures are oriented in north-south direction. ©GoogleEarth 2017

**Table S1.** Linear regression results between logarithmic functional traits (single values) and abiotic environmental factors of 12 T. montanum populations in Central Germany. Riezlern (KW) was excluded due to missing abiotic and biotic environmental factors (F_v_/F_m_, PI, crop height). Blue arrows indicate significant (p<0.05) positive effects and red arrows significant negative effects, whereas their lengths reflect the strength of influence (slope estimate). RH = plant height, AGB = total dry aboveground biomass, LA = leaf area, SLA = specific leaf area, LDMC = leaf dry matter content, F_v_/F_m_ = (variable/maximum) fluorescence, PI = performance index, SPS = stomatal pore surface and SPI = potential conductance index, T_a_= annual temperature, P_a_ = annual precipitation, LAI = leaf area index, CEC_pot_ = potential cation-exchange capacity, N = soil nitrogen content, P = soil phosphor content, K = soil potassium content. *** = p < 0.001.

|  | **H** | **AGB** | **LA** | **SLA** | **LDMC** | **F_v_/F_m_** | **PI** | **SPS** | **PCI** |
| --- | --- | --- | --- | --- | --- | --- | --- | --- | --- |
|  | **[cm]** | **[g]** | **[mm²]** | **[mm²mg^-1^]** | **[mg g^-1^]** |  |  | **[µm²]** |  |
| Model Results | R² = 75%, F_8, 231_ = 88, *** | R² = 57%, F_7, 232_ = 45, *** | R² = 67%, F_10, 229_ = 46, *** | R² = 22%, F_9, 230_ = 7,0, *** | R² = 26%, F_6, 233_ = 14, *** | R² = 21%, F_4, 235_ = 17, *** | R² = 19%, F_8, 231_ = 7,41, *** | R² = 18%, F_6, 233_ = 8,40, *** | R^2^ = 27%, F_8, 231_ = 10, *** |
|  |  |  |  |  |  |  |  |  |  |
| T_a_ |  |  |  |  |  |  |  |  |  |
| P_a_ |  | n.s. |  |  |  |  |  | n.s. |  |
| Slope |  | n.s. |  |  |  |  |  | n.s. |  |
| LAI |  |  |  |  | n.s. | n.s. | n.s. |  | n.s |
| Soil dep. | n.s. | n.s. |  |  | n.s. |  |  | n.s |  |
| CEC_pot_ | n.s. |  |  | n.s. |  | n.s. | n.s. | n.s | n.s |
| pH |  |  |  |  | n.s. | n.s. |  |  |  |
| N |  |  |  |  |  | n.s. |  |  |  |
| P |  |  |  |  | n.s. | n.s. |  |  |  |
| K |  |  |  |  |  | n.s. |  |  |  |

**Table S2.** Mean genetic properties of nine microsatellite markers including 13 T. montanum populations in Central Europe. Allelic size ranges from Matter, Määttänen, Kettle, Ghazoul, & Pluess (2012) are given in brackets. Mean N_A_ = mean allelic richness, P_Ap_ = percentage of populations with private alleles, H_e_ = expected heterozygosity, H_o_ = observed heterozygosity, F_IS_ = inbreeding coefficient, I = Shannon´s diversity index, G_ST_ = differentiation of subpopulations relative to the total population. See also Electronic Supplementary Material Table 4 for microsatellite raw data.

| **Genetic properties** | ***ats006*** | ***ats032*** | ***Tm21*** | ***ats029*** | ***Tm10*** | ***Tm12*** | ***Tm24*** | ***ats002*** | ***Tm16*** |
| --- | --- | --- | --- | --- | --- | --- | --- | --- | --- |
| allelic size range [bp] | 297-321 (303-340) | 129-186 (165-201) | 74-182 (144-188) | 243-252 (267-273) | 137-167 (172-191) | 180-198 (206-215) | 127-217 (156-254) | 206-212 (229-241) | 68-92 (84-114) |
|  |  |  |  |  |  |  |  |  |  |
| mean N_A_ | 4.92 | 10.31 | 12.08 | 2.46 | 4.69 | 3.54 | 11.31 | 2.15 | 7.38 |
| P_Ap_ | 0.00 | 7.69 | 61.54 | 0.00 | 0.00 | 7.69 | 61.54 | 0.00 | 0.00 |
| H_o_ | 0.55 | 0.67 | 0.79 | 0.24 | 0.70 | 0.54 | 0.72 | 0.50 | 0.78 |
| H_e_ | 0.57 | 0.82 | 0.86 | 0.24 | 0.68 | 0.52 | 0.84 | 0.47 | 0.75 |
| I | 1.16 | 2.00 | 2.20 | 0.42 | 1.28 | 0.91 | 2.09 | 0.68 | 1.62 |
| F_IS_ | 0.022 | 0.184 | 0.084 | 0.003 | -0.040 | -0.035 | 0.138 | -0.068 | -0.045 |
| G_ST_ | 0.182 | 0.071 | 0.047 | 0.292 | 0.056 | 0.105 | 0.056 | 0.033 | 0.054 |

**
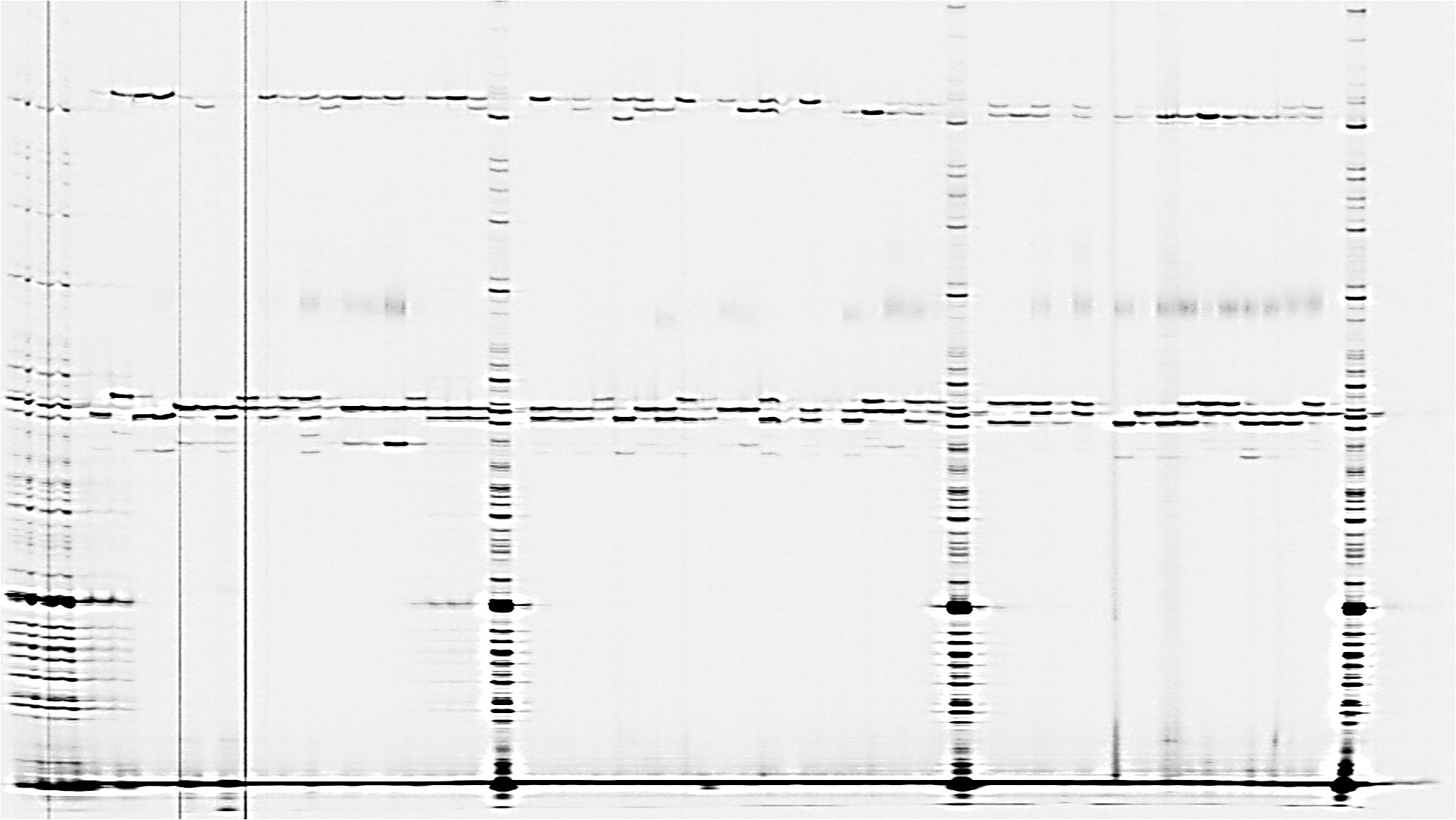
**

**Fig. S3.** Locus ats029 (ATG; 267-273 bp; Matter, Määttänen, Kettle, Ghazoul, & Pluess, 2012) exhibited one of the lowest genetic diversity compared to other microsatellite loci (Table S2). The sizing standard on the left, middle, and right enabled the reviewer to estimate allelic fragment sizes.

**
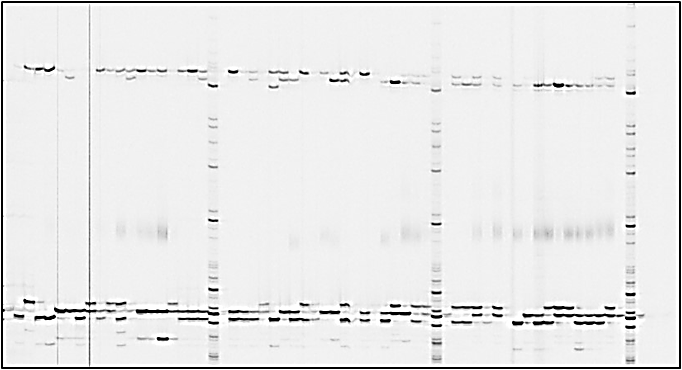
**

**Fig. S4.** Locus Tm10 (GTT; 172-191 bp; Matter, Määttänen, Kettle, Ghazoul, & Pluess, 2012) revealed an average genetic diversity compared to other microsatellite loci (Table S2). The sizing standard on the left, middle, and right enabled the reviewer to estimate allelic fragment sizes.

**
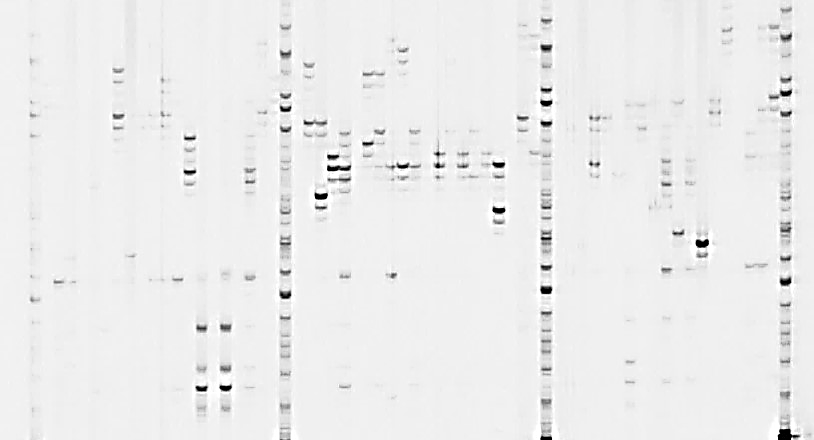
**

**Fig. S5.** Locus Tm21 (GT; 144-188 bp; Matter, Määttänen, Kettle, Ghazoul, & Pluess, 2012) showed one of the highest genetic diversity compared to other microsatellite loci (Table S2). The sizing standard on the left, middle, and right enabled the reviewer to estimate allelic fragment sizes.

**Fig. S6.** A PCoA based on Nei’s genetic distances using genetic data from 255 *T. montanum* individuals (13 populations). Here, we found no evidence of clonality within the sampled populations.

**Table S3.** Results of the path analysis (structural equation model) . We used population size, environmental, genetic, and functional trait information based on 13 locations and up to 260 individuals. Please see Materials and Methods section for more details. CV = coefficient of variation (diversity measure), HD = abiotic habitat heterogeneity (used as environmental diversity index in Karbstein et al., 2020), iFD_CV_ = intraspecific functional trait diversity (measure of ITV; used as diversity index in Karbstein et al., 2020). N_A_ = allelic richness, P_Ap_ = private allelic richness, H_e_ = expected heterozygosity (used as diversity index GD in Karbsteinet al., 2020), H_o_ = observed heterozygosity, F_IS_ = inbreeding coefficient, I = Shannon’s diversity index, G_ST_ = differentiation of subpopulations relative to all populations. *** = p<0.001, ** = p<0.01, * = p <0.05.

| **response** | **predictor** | **estimate** | **standard error** | **degrees of freedom** | **crit value** | **p value** | **standardized estimate** | **explained percentages** | **significance level** |
| --- | --- | --- | --- | --- | --- | --- | --- | --- | --- |
| log(population size) | CV_altitude_ | 209.8 | 49.8 | 4 | 4.22 | 0.013 | 0.601 | 10 | * |
| log(population size) | CV_slope exposure_ | 2.0 | 1.04 | 4 | 1.95 | 0.123 | 0.387 |  |  |
| log(population size) | CV_LAI_ | -26.9 | 4.47 | 4 | -6.04 | 0.004 | -1.437 | 23 | ** |
| log(population size) | CV_soil depth_ | 38.9 | 8.33 | 4 | 4.67 | 0.01 | 1.337 | 22 | ** |
| log(population size) | CV_pH_ | -28.3 | 6.26 | 4 | -4.52 | 0.011 | -0.750 | 12 | * |
| log(population size) | CV_N_ | 14.5 | 3.54 | 4 | 4.11 | 0.015 | 0.774 | 12 | * |
| log(population size) | CV_P_ | -41.1 | 1.62 | 4 | -2.54 | 0.064 | -0.444 |  |  |
| log(population size) | CV_K_ | -29.7 | 5.27 | 4 | -5.63 | 0.005 | -1.309 | 21 | ** |
| HD | log(habitat area) | 0.009 | 0.013 | 11 | 0.71 | 0.493 | 0.209 |  |  |
| N_A_ | log(population size) | 2.16 | 0.763 | 11 | 2.83 | 0.016 | 0.649 |  | * |
| H_o_ | log(population size) | 0.021 | 0.004 | 11 | 4.73 | 0.001 | 0.819 |  | *** |
| H_e_ (GD) | log(population size) | 0.010 | 0.004 | 10 | 2.45 | 0.034 | 0.584 |  | * |
| H_e_ (GD) | HD | 0.110 | 0.108 | 10 | 1.02 | 0.332 | 0.243 |  |  |
| I | log(population size) | 0.037 | 0.009 | 11 | 4.01 | 0.002 | 0.771 |  | ** |
| F_IS | log(population size) | -0.015 | 0.006 | 11 | -2.42 | 0.034 | -0.590 |  | * |
| G_ST_ | log(population size) | -0.002 | 0.001 | 11 | -1.67 | 0.123 | -0.450 |  |  |
| iFD_CV_ | HD | 0.303 | 0.065 | 10 | 4.70 | 0.001 | 0.707 | 68 | *** |
| iFD_CV_ | H_e_ (GD) | 0.319 | 0.142 | 10 | 2.24 | 0.049 | 0.338 | 32 | * |

**References**

Karbstein, K., Prinz, K., Hellwig, F., & Römermann, C. (2020). Plant intraspecific functional trait variation is related to within‐habitat heterogeneity and genetic diversity in *Trifolium montanum* L. *Ecology and Evolution*, *10*(11), 5015–5033. doi:10.1002/ece3.6255

Matter, P., Määttänen, K., Kettle, C. J., Ghazoul, J., & Pluess, A. R. (2012). Eleven microsatellite markers for the mountain clover *Trifolium montanum* (Fabaceae). *American Journal of Botany*, *99*(11), 447–449. doi:10.3732/ajb.1200102
